# Systematic multi-reference vertebrate ACE2 sequence similarity analysis predicts species susceptibility to SARS-related sarbecoviruses

**DOI:** 10.1038/s41598-026-41410-9

**Published:** 2026-03-18

**Authors:** John A. Frank, Eric Xu Gan, William Brenham Hooper, Isabel Madeleine Ott, Akiko Iwasaki

**Affiliations:** 1 Department of Immunobiology, Yale University School of Medicine, New Haven, CT 06520 USA; 2 Center for Infection and Immunity, Yale University School of Medicine, New Haven, CT 06520 USA; 3 Department of Molecular, Cellular and Developmental Biology, Yale University, New Haven, CT 06520 USA; 4 Howard Hughes Medical Institute, Chevy Chase, MD 20815 USA; 5 Present Address: Department of Biology, Linfield University, McMinnville, OR 97128 USA; 6 Present Address: Department of Genetics, University of North Carolina, Chapel Hill, NC 27517 USA

**Keywords:** SARS-CoV-2, ACE2, Receptor, Animal susceptibility, Sequence, Infection, Bioinformatics, Data integration, Data mining, SARS-CoV-2, SARS virus, Viral transmission, Virus-host interactions

## Abstract

SARS-CoV-2 infection of humans and its spillover into new species illustrates the need for effective predictive tools designed to identify host ranges of emerging zoonotic viruses. We hypothesized that host receptor sequence similarity across animals could be leveraged to identify species at risk for cross-species virus transmission. We developed a flexible computational pipeline, **M**ulti-**r**eference **S**imilarity **A**nalysis of **R**eceptor **S**equences (MrSARS), to compare homologous virus-binding receptor sequences to multiple reference host sequences. MrSARS applies an aggregate similarity score to each examined receptor sequence and categorizes them as highly or moderately susceptible, or resistant to infection. We used the sarbecovirus receptor angiotensin converting enzyme 2 (ACE2) as a model to test MrSARS predictions because ACE2 has been extensively sequenced, and its interaction with SARS-CoV and SARS-CoV-2 glycoproteins characterized. We analyzed 825 vertebrate ACE2 sequences and determined that primates and even-toed ungulates ranked highest among susceptible species. We tested these predictions by infecting 293T cells, transiently expressing ACE2 from confirmed, putatively susceptible, and resistant species, with SARS-CoV-2 variant and SARS-CoV-related glycoprotein-pseudotyped VSV reporter viruses. These experiments correlated with MrSARS susceptibility predictions and existing literature. Our study illustrates that receptor sequence information from multiple susceptible species can identify potential host ranges of circulating viruses.

**Supplementary Information:**

The online version contains supplementary material available at 10.1038/s41598-026-41410-9.

## Introduction

Virus transmission within and between species can have significant economic and public health consequences. Zoonotic viruses, which jump from animals to humans^1^, have caused the most impactful human epidemics (e.g., SARS-CoV-2, Influenza A virus (IAV), HIV, SARS-CoV, Ebola) in recent years^2^. Viruses can also transmit from humans back into animals, and between wild and domestic animal populations. Virus spillover into domesticated farm animals can cause severe economic and agricultural disruptions^2^. In the past two decades, SARS-CoV-2, IAV and Henipavirus spillover events have forced farmers to cull vast domestic mink, fowl, and swine populations respectively^3–5^.

Despite the proven efficacy of wildlife conservation, public health infrastructure improvements, animal handling regulations, and proactive virus surveillance in managing virus spillover events^6^, these interventions are often hampered by underfunding^6,7^. Surveillance resources typically target species known or suspected to harbor viruses with zoonotic potential, historically focusing on bat, rodent, and bird populations. Bats and rodents are sensible surveillance targets because these animals are the two most species-rich mammalian lineages^8^. However, focusing solely on these animals, based on previous pandemics, may miss novel reservoirs or intermediate zoonotic virus hosts. Less biased and user-friendly strategies to identify susceptible host species for a given pathogen can improve targeting strategies. We hypothesize that virus receptor sequence analysis of diverse and known susceptible animals can be used to home in on potentially important virus spillover targets. Such analyses, when combined with existing experimental and animal surveillance research, can help field scientists and public health officials prioritize their virus surveillance and animal management strategies.

Several studies have sought to predict the SARS-CoV-2 host range by analyzing sequence similarity relative to humans, calculating virus binding energy, employing machine learning tools, experimentally testing subsets of species, or employing some combination of these methods (Table S1). Each approach has its strengths and weaknesses. Thus far, sequence-based ACE2 analyses have tended to define animal susceptibility to SARS-CoV-2 only in reference to humans. While this approach is simple and can be accurate for species that share strong residue identity with humans, these analyses overlook information that can be gleaned from non-human species (such as white-tailed deer, mink, and mouse) with known susceptibility to infection by ancestral and variant SARS-CoV-2 strains. Analyses of binding energies between predicted animal ACE2 protein structures and a single SARS-CoV2 spike structure suffer from similar limitations because they limit the ascertained binding energies to a putative interaction of animal ACE2 and one specific SARS-CoV-2 spike variant or sarbecovirus spike. Thus, this approach is not broadly applicable to the analysis of SARS-CoV-2 variants or related sarbecoviruses because the structural interaction and associated binding energy is exclusively limited to the characterized ACE2 and spike structure. It must also be noted that binding energy is not a perfect predictor of virus entry. Several experimental studies have shown that spike-ACE2 binding affinity does not directly correlate with cell entry^9–12^. Machine learning approaches are promising for integrating varied data types including animal ACE2 sequence similarity, phylogenetic information, geographic distribution, and genomics^13–16^. However, these tools tend to (i) be computationally intensive, (ii) require extensive expertise to reliably construct and operate, and (iii) be opaque in terms of reporting what factors are leading to a model’s predictions. While using full-length, partial, or chimeric spike proteins to test spike-dependent receptor binding and/or cell entry in vitro is a powerful tool to explore host range^17,18^, synthesizing host receptor and spike plasmids can be cost-prohibitive for many research groups. Thus, experimentalists require robust tools to prioritize receptor subsets for testing.

There are numerous molecular host tropism determinants. A successful spillover infection requires that a virus successfully utilize host machinery at all stages of the virus replication cycle^19^. These factors range from receptor binding and virus entry to virus replication, and release. Receptor protein binding and cell entry represents the first molecular barrier to infection of a new host species for several viruses^19^**.** Variation at the receptor-virus binding interface can dramatically affect the probability of successful cell entry^19^**.** Because a virus cannot proceed through its life cycle without cell entry, examining the feasibility of virus entry is a logical first step in identifying candidate species that may be susceptible to spillover infections. Here we developed a receptor sequence analysis method that builds on existing receptor sequence-based host susceptibility predictions by including receptor sequences from non-human hosts. Using the SARS-CoV-2 and related sarbecoviruses receptor ACE2 as a model, we developed a user friendly and robust **M**ulti-**r**eference **S**imilarity **A**nalysis of **R**eceptor **S**equences (MrSARS) pipeline that principally utilizes receptor protein sequence similarity and known animal susceptibility to analyze and quantify putative species susceptibility. Our pipeline identifies a subset of putatively susceptible species comparable in number and species identity to previous studies. While our approach relies on pre-existing knowledge of virus-bound receptor residues, which can be determined by various experimental methods (including genetic, biochemical, or structural experiments), this approach does not require pre-existing knowledge of virus-receptor complex structures. MrSARS can be flexibly applied to receptor protein sequences for viruses that have been shown to infect phylogenetically diverse animal lineages (e.g., SARS-CoV-2) or appear to have more phylogenetically restricted host tropism (e.g., SIV). Our approach accounts for phylogenetic diversity among susceptible species by including representative reference sequences from distinct mammalian lineages where SARS-CoV-2 infection has been observed. The choice of reference sequences will depend on the researcher’s virus- or host-specific question. Our pipeline provides a quantitative readout for prediction quality and a host susceptibility ranking, which can be used to prioritize surveillance and intervention efforts.

## Results

### Use of ACE2 sequence similarity to elucidate species susceptible to sarbecovirus infection.

To perform our receptor similarity analysis, we assembled a comprehensive dataset of ACE2 peptide sequences by searching NCBI peptide and nucleotide databases, previous publications, and assembled ACE2 open reading frames (ORF) from a subset of animal genome assemblies^20–23^. Because ACE2 allelic variation in Rhinolophus (horseshoe) bats can affect susceptibility to sarbecoviruses^24,25^, we included all available, high-quality vertebrate ACE2 alleles to account for the possibility that species may harbor non-reference alleles that are susceptible to sarbecovirus binding. ACE2 ORF sequences were processed to retain only virus-bound residues as previously defined by Damas et al. (2020). Following this step, duplicate sequences were removed resulting in a final dataset of 825 unique virus-targeted ACE2 sequences (Fig. S1a). Mammals (49.9%, n = 412), birds (14.9%, n = 123), and ray-finned fish (27%, n = 223) comprised the majority of analyzed ACE2 sequences (Fig. S1b). Bats encompassed approximately one-quarter of assembled mammalian ACE2 sequences (n = 112). To our knowledge, this dataset represents the most comprehensive analysis of ACE2 sequences to date.

**Fig. 1:**
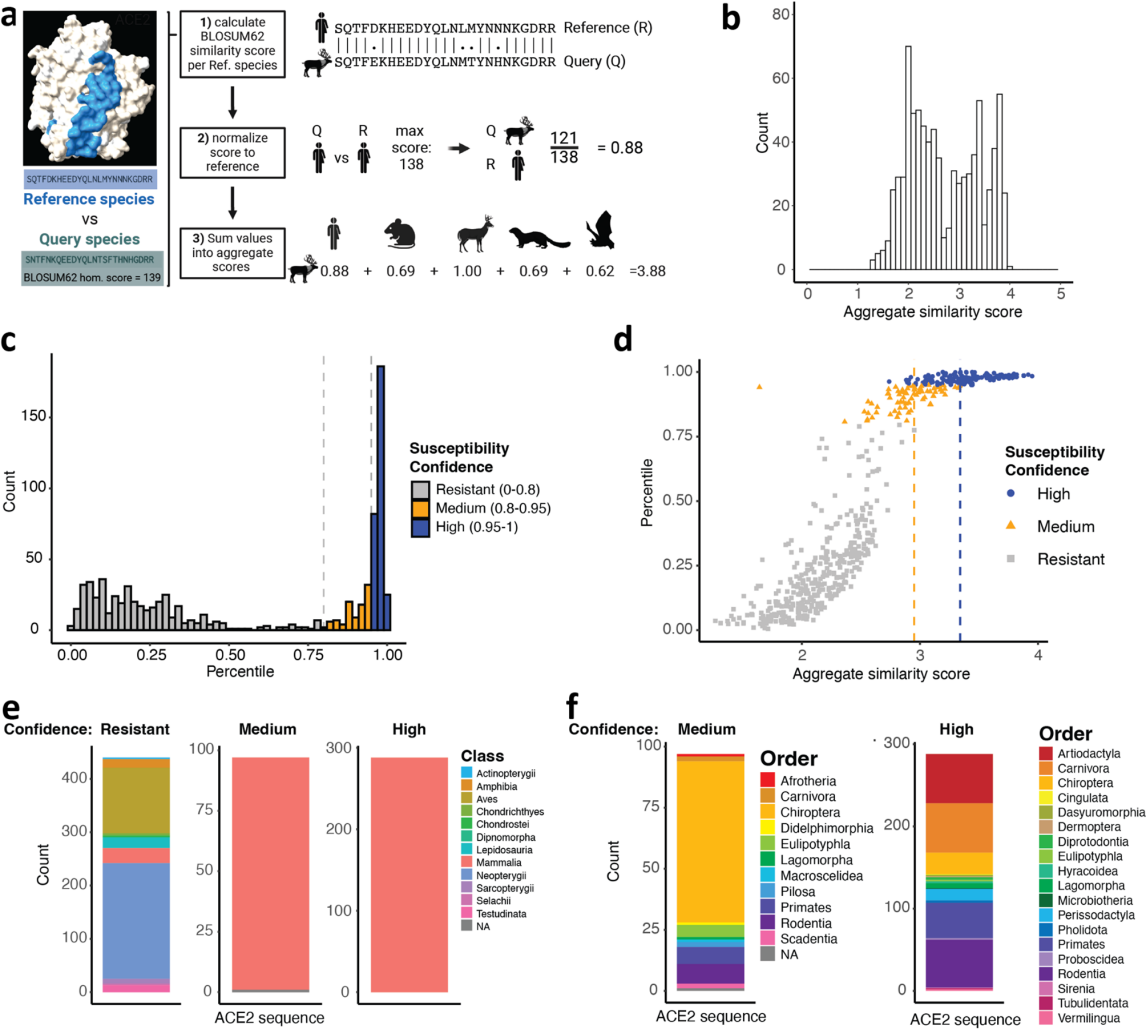
Multi-reference ACE2 sequence homology analysis identifies putatively susceptible species. (**a**) ACE2 sequence homology analysis pipeline outline. Query sequences are individually compared to reference sequences: human, mouse, white-tailed deer, mink, and intermediate horseshoe bat. (**b**) Species aggregate score distributions using bona fide susceptible reference species. (**c**) Distribution of query animal aggregate score percentiles following 1000 × bootstrapped homology pipeline execution using randomly sampled reference species across each bootstrap. Bars are colored according to susceptibility confidence. (**d**) Scatter plot of query sequence percentiles vs aggregate similarity score. Points are colored according to susceptibility confidence defined in (**c**). (**e**, **f**) Animal class (**e**) and order (**f**) representations within high and medium confidence susceptible, and putatively resistant ACE2 sequences.

We passed this ACE2 dataset through our MrSARS pipeline. MrSARS takes our processed ACE2 sequences as inputs along with a list of predefined reference sequences included in the input file. MrSARS uses the BLOSUM62 substitution matrix^26^ to calculate pairwise sequence similarity scores for each query relative to each provided reference sequence (Fig. 1a). These scores are then min–max normalized using the maximum possible score for each reference, producing similarity values ranging from 0 (no similarity) to 1 (perfect sequence similarity). Normalized similarity scores against all reference sequences are summed into an **A**ggregate **S**imilarity (AS) score ranging from 0 to the number of provided reference sequences. For our ACE2 analysis, we chose reference sequences based on the following criteria: (i) The chosen sequence was shown to be susceptible to ancestral SARS-CoV-2 or a variant of concern. (ii) These sequences are representative of species from distinct mammalian lineages. We used reference ACE2 sequences from *Homo sapiens* (human) *Mus musculus* (mouse), *Odocoileus virginianus* (white-tailed deer), *Neogale vison* (American mink) and *Rhinolophus affinis* (intermediate horseshoe bat). Human, mink, and intermediate horseshoe (affinis) bat were chosen because these species or their receptor were reported to be susceptible to the ancestral SARS-CoV-2 strain^27–29^. Mouse and white-tailed deer were included because these species were found to be susceptible to SARS-CoV-2 variants^30,31^. Our pipeline generated AS scores ranging from 1.27 to 3.95 (Fig. 1b). ACE2 sequences from catarrhine primates, aquatic and terrestrial artiodactyls and feline carnivores, occupied the top 10% of all analyzed sequences (Table 1, S1).

**Table 1:**
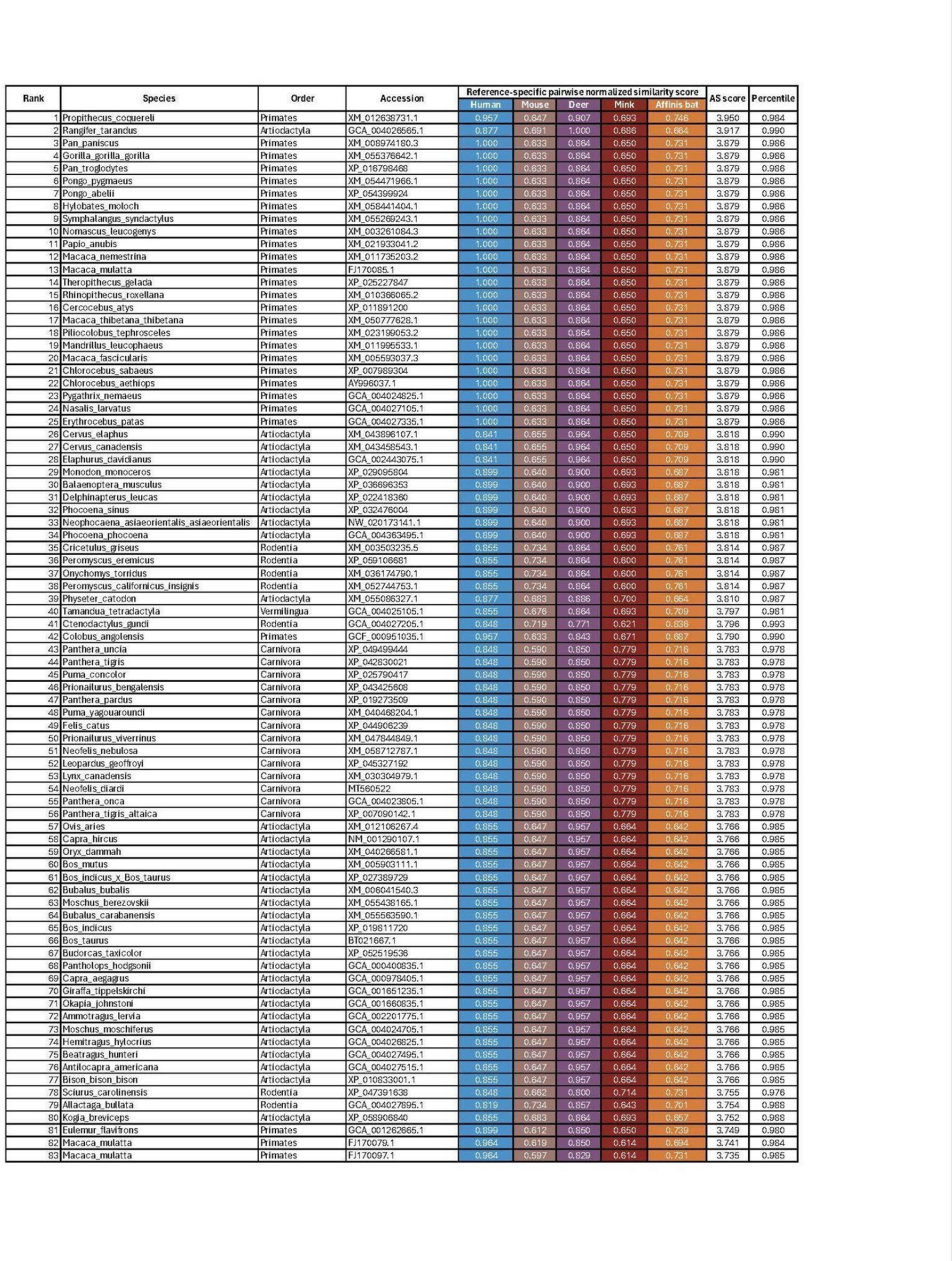
MrSARS pairwise and aggregate similarity scores for top 10% of analyzed sequences.

To define which AS scores reflect meaningful similarity, we determined threshold values to represent confidence. We bootstrapped our pipeline 1000 times using randomly sampled reference species, generating an AS score distribution for each query species. We then examined where on the distribution our true AS score (i.e., generated using true reference species) fell. AS scores were categorized as ‘high confidence’ if they fell above the 95th percentile, and ‘medium confidence’ if they fell within the 80th-95th percentile; all remaining scores were categorized as ‘putatively resistant’ (Figs. 1c, S2). Notably, AS scores from our medium and high confidence groups were distinctly higher compared to sequences falling within the resistant category (Fig. 1d). Any AS score above 2.74 fell exclusively within the high confidence category (Fig. 1d). We found that non-mammalian vertebrates fall within the putatively resistant category (Figs. 1e, S3). Our high and medium confidence susceptibility categories exclusively contained mammals (Fig. 1e). Mammalian orders were relatively evenly represented among sequences with high confidence susceptibility. Primates (14.9%), carnivores (20.8%), rodents (20.1%), artiodactyla (20.8%), and bats (9%) encompass most high-confidence sequences (Fig. 1f). At 68% (66/97), bat sequences tended to dominate the medium confidence group. Microbats, which encompass sarbecovirus-harboring Rhinolophus (horseshoe) and Hipposideros (roundleaf) bats^32^, made up the vast majority (89.4%, 59/66) of bat sequences within the medium confidence group (Fig. 1f). In the high confidence category, microbat ACE2 sequences (19/26) were twice as abundant as megabat (7/26). Based on these observations, MrSARS could generate an easily interpretable similarity value that allows us to rank species. Bootstrapping MrSARS using randomly sampled reference sequences further allowed us to categorize sequences into easily interpretable susceptibility confidence groups.

### MrSARS prediction of animal susceptibility to SARS-CoV-2 and related ACE2-utilizing sarbecoviruses

**Fig. 2:**
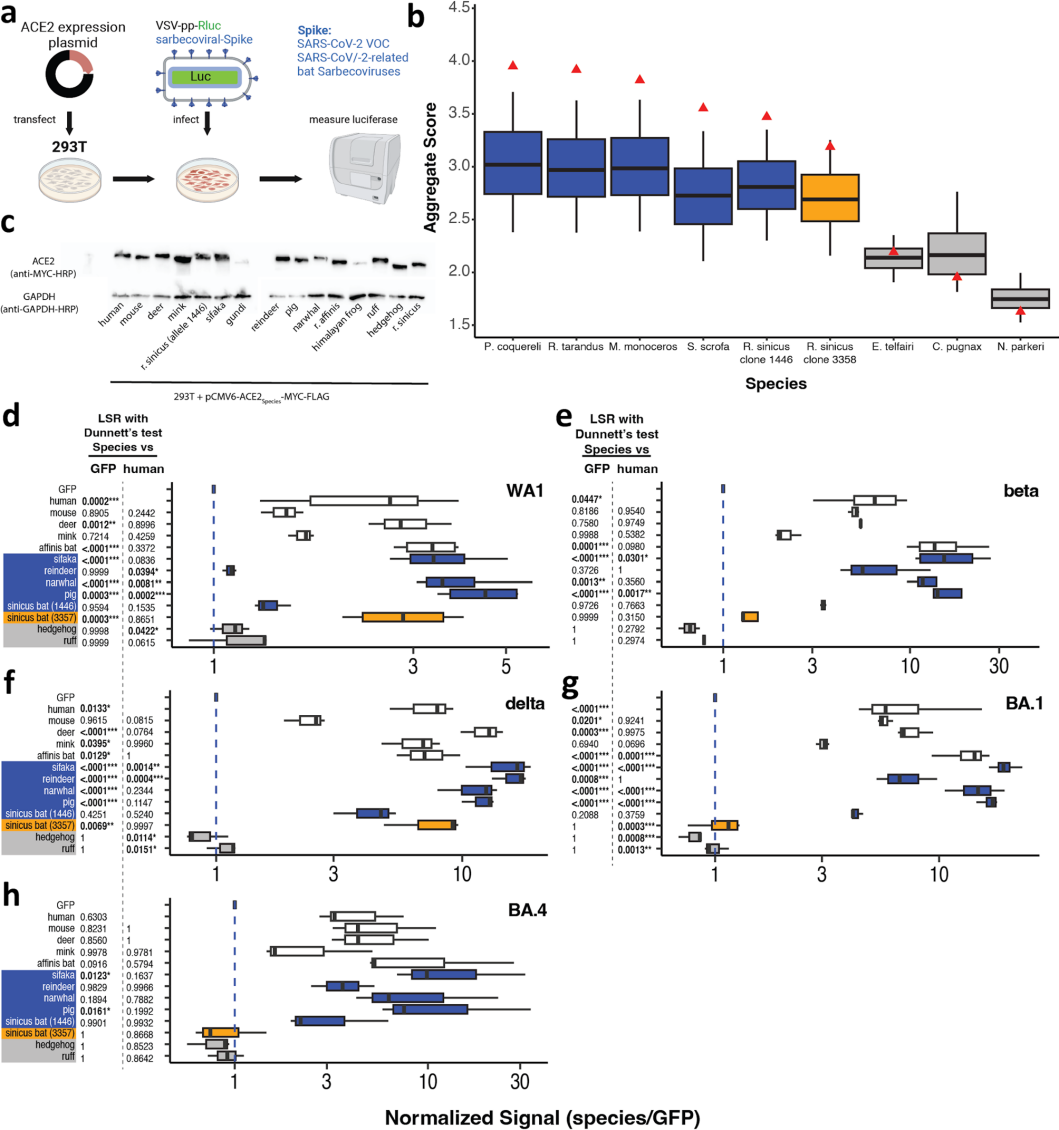
ACE2 from putatively susceptible species permit SARS-CoV-2 spike-dependent infection. (**a**) Infection assay methodology using transiently expressed ACE2 and spike-pseudotyped VSV reporter virus. (**b**) True (red triangle) vs distribution of bootstrapped (box plot) aggregate scores for indicated ACE2 sequence species. Boxplots are colored according to susceptibility confidence defined in Fig. 1. True (red), 95th percentile cutoff, and median (Med) values are shown above each plot. (**c**) Cropped representative Western Blot of 293T cells transiently expressing ACE2 from indicated species. d-h) Normalized infection rates of cells transfected with ACE2 from indicated species and infected with SARS-CoV-2 WA1 (**d**), beta (**e**), delta (**f**), BA1 (**g**), and BA4 (**h**) spike-pseudotyped reporter viruses. Data were analyzed by Least Squares Regression (LSR) with Dunnett’s test; n ≥ 3 with ≥ 2 technical replicates; ***adjusted *p* < 0.001, **adjusted *p* < 0.01, and *adjusted *p* < 0.05.

To experimentally test MrSARS predictions, we used a well-established infection reporter system^33,34^ where coronavirus spike proteins decorate replication-deficient vesicular stomatitis virus (VSV) particles encoding a Renilla luciferase reporter (VSVpp-Rluc). 293T cells transiently expressing C-terminally MYC-FLAG-tagged ACE2 were challenged with SARS-CoV-2 spike-pseudotyped VSVpp-Rluc reporter viruses, and the degree of infection was measured by luciferase assay (Fig. 2a). 293T cells transiently expressing GFP served as an ACE2-independent infection control.

We tested our reference species (human, mouse, white-tailed deer, mink, affinis bat) and a subset of ACE2 sequences to represent all assigned categories and overall rankings (Fig. 2b). Coquerel’s sifaka (AS rank: 1), reindeer (AS rank: 2), narwhal (AS rank: 29), gundi (AS rank: 43) pig (AS rank: 142), and Chinese horseshoe (sinicus) bat allele 1443 (AS rank: 150) represent high confidence susceptible species. Sinicus bat allele 3358 (AS rank: 262) served as a medium confidence ACE2 sequence. Hedgehog (AS rank: 579), and ruff (rank: 719), and Himalayan frog (rank: 805) were included as putatively resistant ACE2 sequences. To ensure robust and comparable ACE2 protein expression (Fig. 2c), we performed Western blots of 293T cells transiently expressing ACE2 from above-described species. Except for the Himalayan frog and gundi, we found that ACE2 from all other species was robustly expressed at similar levels. Consequently, Himalayan frog and gundi ACE2 were excluded from all following experiments. We assessed protein folding and function using angiotensin conversion assays and found that all tested proteins are enzymatically active (Fig. S4) indicating ACE2 is likely folding properly and thus likely to be present at the cell surface.

ACE2 expressing 293T cells were challenged with VSVpp-Rluc pseudotyped with ancestral SARS-CoV2 spike (WA1-S) (Fig. 2d). We found that human, deer and affinis bat ACE2 were significantly susceptible to infection mediated by WA1-spike above background. While both mouse and mink ACE2 trended towards mediating WA1-S dependent virus entry above background, this difference was not statistically significant. Mouse and mink ACE2’s weaker susceptibility to WA1-S is consistent with existing literature indicating these receptors mediate WA1 cell infection at lower rates^23,28,35,36^. Cells expressing hedgehog and ruff ACE2 were not infected above background, indicating that these ACE2 proteins were resistant to WA1-S-mediated entry. ACE2 from sifaka, narwhal, pig, and sinicus bat allele 3358 all showed infection rates comparable to or higher than our reference species. Contrary to MrSARS predictions, reindeer and sinicus bat allele 1443 were largely resistant to WA1-S-mediated infection. In summary, MrSARS’ susceptibility predictions were experimentally supported for 6 out of 8 susceptibility predictions using WA1-S-mediated infection as our reporter.

The AS score ranking generated by MrSARS used reference sequences of species that were susceptible to the ancestral (human, mink, affinis bat) strain or variant (mouse, affinis bat, white-tailed deer) strains of SARS-CoV-2. Thus, ACE2 from our putatively susceptible species may mediate virus entry for a subset of variants. To test this possibility, we challenged 293T cells transiently expressing ACE2 from the above-described species with VSVpp-Rluc pseudotyped with spike from SARS-CoV-2 Beta, Delta, and Omicron (BA.1 and BA.4 sublineages) variants of concern (VOC) respectively (Fig. 2e–h). As with WA1-S, reference species were generally susceptible to all variants. Due to variability in VSVpp-Rluc pseudotyping efficiency, the infection signal above the GFP background could not be confirmed for all tested reporter viruses. With this caveat in mind, we made the following observations. Mouse ACE2 was a relatively poor receptor for delta-S but as susceptible as human for all other tested VOC spikes. Mink ACE2 was highly susceptible to delta-S and BA1-S but less so for beta-S and BA4-S. Deer and affinis bat ACE2 proteins were universally susceptible to VOC-S-dependent virus entry at equivalent or higher levels than human ACE2. No putatively resistant species were susceptible to VOC spike-mediated infection above background. Except for reindeer ACE2 (Fig. 2d), high-ranking species within the high-confidence group tended to be susceptible to infection mediated by all VOC-S at levels comparable to or surpassing human ACE2. Lower-ranking species within the high-confidence group and the medium-confidence group showed strain specific variability. Sinicus bat 1443 ACE2 was susceptible to beta-S, delta-S, BA.1-S, and BA.4-S but less so for WA1-S. Conversely, sinicus bat 3358 ACE2 was susceptible to WA1-S and delta-S but resistant to beta-S, BA.1-S, and BA.4-S.

Our infection data were generally consistent with MrSARS predictions. High confidence ACE2 sequences were largely susceptible to tested pseudoviruses while putatively resistant ACE2 sequences did not mediate infection. Sinicus bat 3358 ACE2 showed spike-specific susceptibility, which correlates with its medium confidence categorization. Variable species susceptibility indicated that human-specific SARS-CoV-2 evolutionary adaptation combined with animal sequence variation at SARS-CoV-2 spike-bound ACE2 residues is likely to affect the range of species susceptible to spillover events from humans.

**Fig. 3:**
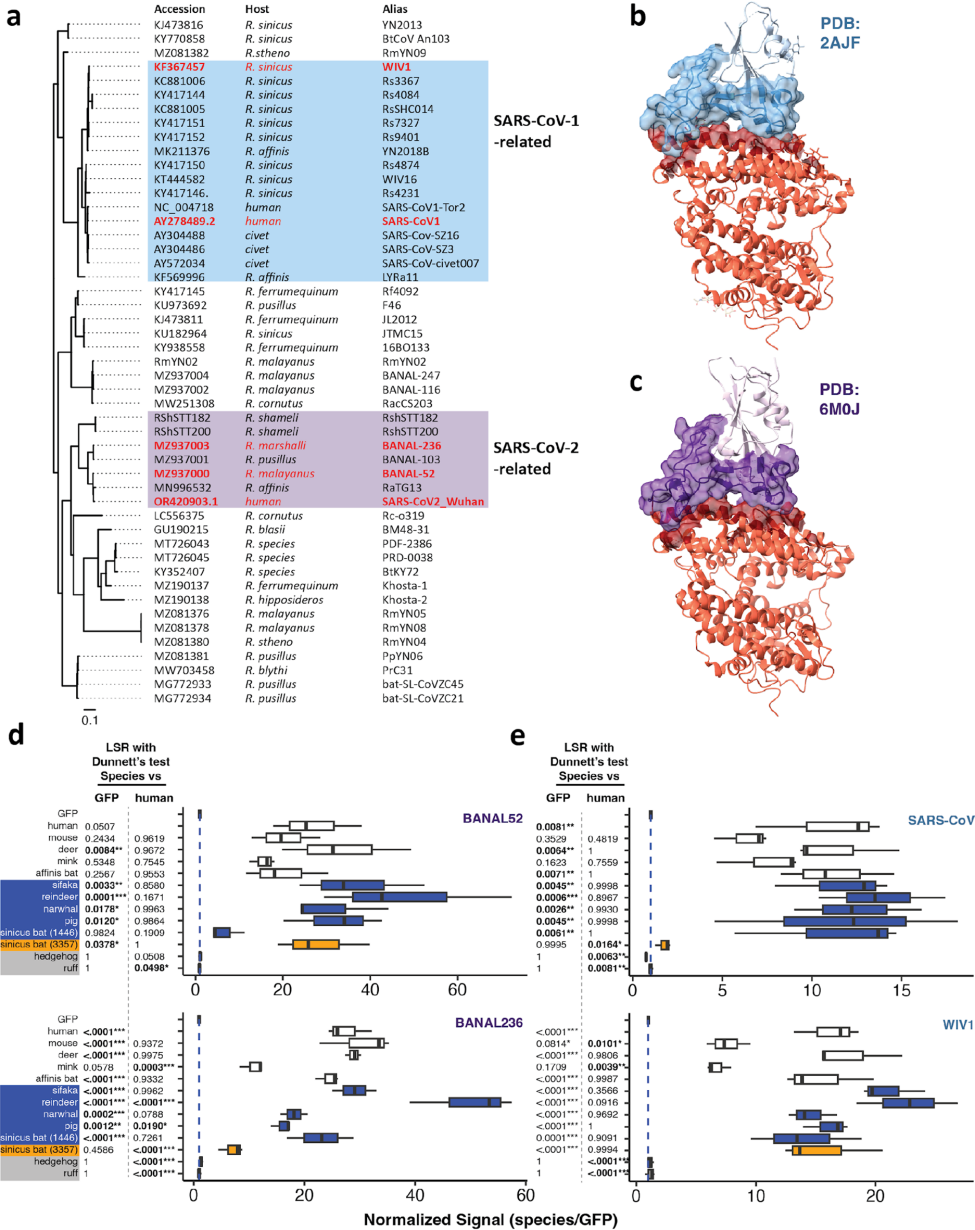
ACE2 infection susceptibility extends to SARS-CoV/SARS-CoV-2-related sarbecoviruses. (**a**) Phylogenetic tree of ACE2-utilizing sarbecoviruses. Tree generated based on spike sequence alignment. Experimentally tested spikes are highlighted in red. (**b**, **c**) Crystal structures from indicated PDB files of human ACE2 (red) bound to SARS-CoV (**b**) and SARS-CoV-2 (**d**) spike respectively. ACE2-spike interacting residues and spike receptor binding motifs are shaded darker and illustrated with transparent surface models. (**d**) Normalized infection rates of cells transfected with ACE2 from indicated species and infected with SARS-CoV-2 (**d**) and SARS-CoV (**e**) -related sarbecovirus spike-pseudotyped reporter viruses. Data were analyzed by Least Squares Regression (LSR) analysis with Dunnett’s test; n ≥ 3 with ≥ 2 technical replicates; ***adjusted *p* < 0.001, **adjusted *p* < 0.01, and *adjusted *p* < 0.05.

Several SARS-CoV and SARS-CoV-2-related sarbecoviruses have been reported to utilize ACE2^17,28,32,37,38^ (Fig. 3a). Many of these viruses utilize the same binding interface as SARS-CoV and SARS-CoV-2^12,17,18,39,40^ (Fig. 3b, c). Thus, we hypothesized that our susceptibility predictions should extend to SARS-CoV and SARS-CoV-2-related sarbecoviruses. We replicated the above infection assays with VSVpp-Rluc reporter viruses pseudotyped with SARS-CoV-2 related spikes from BANAL-52 and BANAL-236. We also assessed the ability of our ACE2 constructs to mediate entry by spike from SARS-CoV and its relative, WIV1.

As with SARS-CoV-2, ACE2 from all tested reference species mostly supported reporter virus entry by all tested sarbecovirus spikes (Fig. 3d, e). Human, deer, and affinis bat ACE2 supported generally comparable infection rates across all tested sarbecovirus spikes. Mouse and mink ACE2 were less efficiently utilized by SARS-CoV-related spikes. BANAL-236 utilized mink ACE2 less efficiently than ACE2 from other reference species. ACE2 from putatively resistant species did not mediate sarbecovirus spike-dependent entry above GFP background. Excluding sinicus bat 1443 ACE2, all other tested high-confidence species were susceptible to sarbecovirus spike-mediated infection above GFP background. Under most circumstances, cells expressing ACE2 from these species were infected at rates comparable to our reference species. However, virus entry efficiency can vary by species and virus strain. Notably, reindeer ACE2 was significantly more susceptible to BANAL-236 compared to our reference species. Sinicus bat ACE2 alleles 1443 and 3358 exhibited virus-specific susceptibility. Sinicus 1443 was resistant to BANAL-52 spike-mediated virus entry, but susceptible to all other tested sarbecovirus spikes. Conversely, sinicus 3358 was susceptible to BANAL-52 and WIV1 but resistant to BANAL-236 and SARS-CoV spike-mediated infection. This variable susceptibility across sinicus alleles correlates with sinicus bat’s known status as a SARS-CoV and SARS-CoV-2 related sarbecovirus reservoir^32^. Our data for affinis bat and sinicus bat allele 3358 were corroborated by Fujita et al. using the identical sinicus allele and a closely related (99% peptide sequence identity) affinis allele^41^. ACE2 is known to have been subject to extensive positive selection in sinicus bats, resulting in the emergence of ACE2 alleles that are differentially susceptible to various SARS-CoV-related sarbecoviruses^24^.

### MrSARS ACE2 predictions in the context of existing literature

To assess whether our pipeline’s predictions are consistent with previous reports, we searched the literature for studies on SARS-CoV/SARS-CoV-2-related sarbecovirus use of non-human ACE2 or their detection in animals. SARS-CoV/SARS-CoV-2 ancestral strains and variants, and SARS-CoV/SARS-CoV-2-related viruses were included in our analysis to account for their usage of common ACE2 residues. Our final dataset included 115 studies published from 2003 to 2024 (Table S1), encompassing 5640 distinct vertebrate species and 83 distinct SARS-CoV/SARS-CoV-2-related sarbecoviruses (Fig. S5). The number of host species included per study ranged from 1 to 275 species, with two outlier computational studies^16^ examining 5400 and 409^23^ unique species respectively (Fig. S6). To differentiate between the types of data generated in these studies categorized as in silico, in vitro, in cellus, or in vivo (Fig. S7). In silico studies included ACE2 sequence similarity, phylogenetics, structure, or hybrid sequence similarity/structure susceptibility predictions (n = 5626). These predictions exclude experimentally generated data. Studies experimentally measuring spike-ACE2 binding, but not virus entry, were categorized as in vitro (n = 121). Studies experimentally measuring viral entry of cultured cells via ACE2 were classified as in cellus (n = 167). These studies must have been conducted in a cell culture system and can have employed sarbecoviruses or spike-pseudotyped reporter viruses. Studies where viral infection was detected in whole organisms were classified as in vivo (n = 68). Seropositivity, PCR-based detection, virus titer, and experimental infection were included in this category.

To directly compare our MrSARS predictions to published studies, we categorized species in our meta-analysis into three categories: susceptible, ambiguous, or resistant to SARS-CoV/SARS-CoV-2-related sarbecovirus infection (see methods). We then intersected the species from our literature dataset with the species identities of receptor sequences analyzed with MrSARS. This yielded 528 unique species that were shared between the literature dataset and MrSARS (Table S2), with 181 species unique to MrSARS (Fig. S8a), which are primarily fish, birds, and mammals (Fig. S8b). We first examined how consistent animal susceptibility predictions/reports are across published studies and found that 67% of analyzed species (398/533) have perfect consistency across studies. Notably, all confirmed in vivo infections were in species predicted to be susceptible with high or medium confidence by MrSARS (Fig. S9). This left 33% (172/528) with conflicting reports of SARS-related sarbecovirus susceptibility. Bats (n = 59, 34.3%), carnivores (n = 31, 18%), primates (n = 28, 16.3%), artiodactyls (n = 35, 20.3%) and rodents (n = 23, 13.4%) were the major animal orders that contributed to this inconsistency across study types (Fig. S9). In vitro and in cellus studies tended to exhibit the greatest species-specific discrepancies across non-SARS-CoV-2 virus classes (Fig. S10, S11). The machine learning approach described by Fischhoff et al.^16^ (n = 5400) and combined sequence and structure prediction-based analysis by Damas et al.^23^ (n = 409) each included a disproportionately large fraction of species in our dataset. Thus, we directly compared MrSARS confidence categories to the Fischhoff and Damas studies respectively to avoid biasing our meta-analysis of the in silico dataset.

We assessed the chance-corrected degree of ordinal agreement between MrSARS and our meta-analysis categories by calculating weighted Cohen’s Κ and Krippendorf’s ɑ (Table S3). Cohen’s K analyses revealed a statistically significant agreement across all examined categories apart from the in vivo dataset. However, MrSARS predictions were only in substantial agreement with the in silico literature (K = 0.7252) and show fair agreement with Damas (K = 0.3765) and the in vitro (K = 0.4025) literature^42^. The Fischhoff study (K = 0.07719) and in cellus (K = 0.08485) literature showed only slight agreement with MrSARS’ predictions. Krippendorf’s ɑ statistics largely matched Cohen’s K statistics, though with higher stringency. MrSARS’ predictions could only be classified as having acceptable agreement with the in silico literature (ɑ = 0.698, CI: 0.61—0.77)^43^. All other comparisons fell below ɑ = 0.667, the canonical minimum threshold for agreement between two methods.

**Fig. 4:**
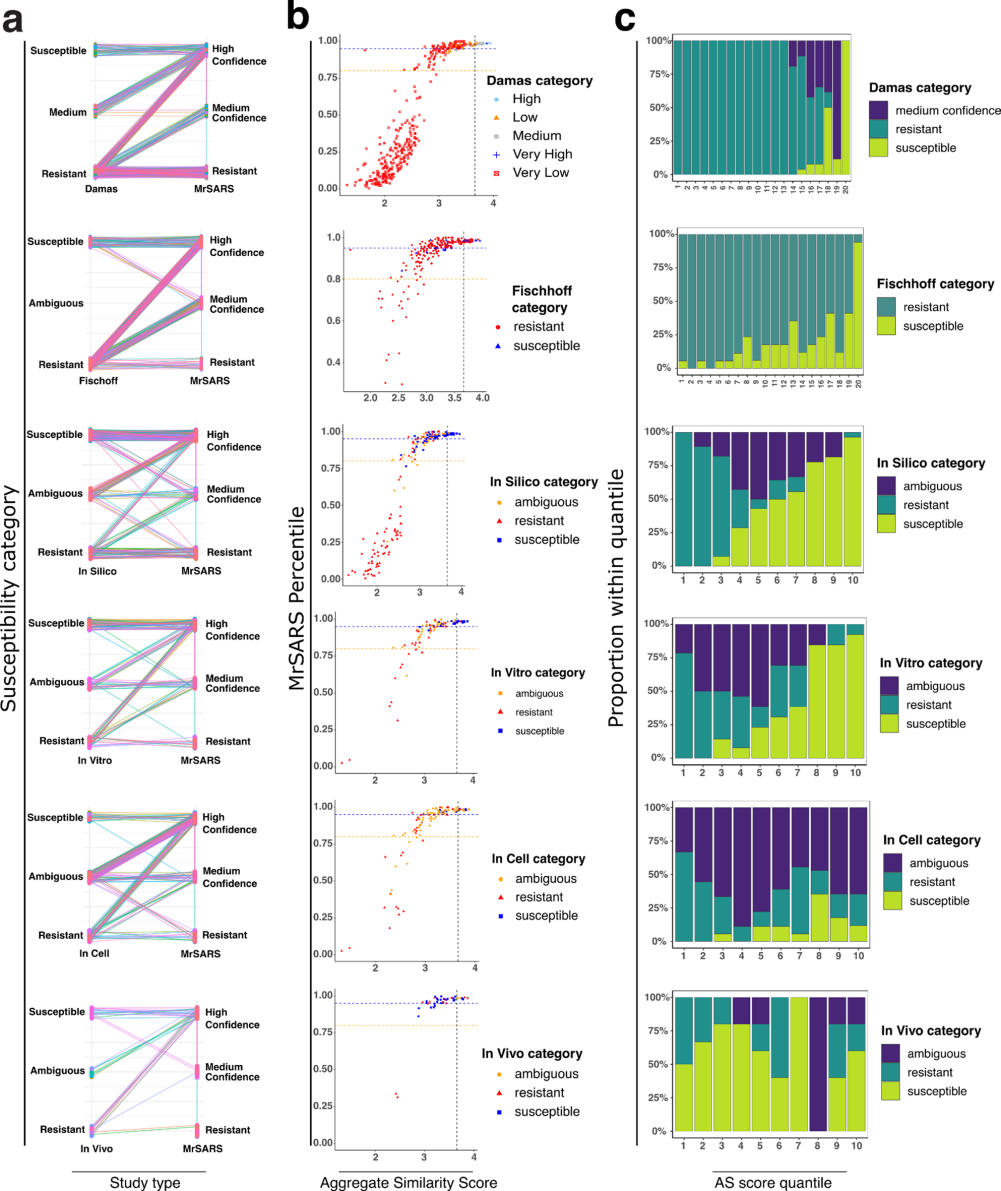
Comparison of literature vs MRSARS pipeline predictions. (**a**) Connected scatter plots depict species-specific susceptibility categorizations. Jittered dots connected by lines represent each individual species compared between the indicated published study type and MrSARS. (**b**) Scatter plot of MrSARS sequence percentiles vs aggregate similarity score. Points are representative of species examined by indicated study types and colored according to susceptibility as defined in our meta-analysis. Horizontal dashed lines denote the cutoff point for MrSARS 95th (blue) and 80th (orange) percentiles. (**c**) Stacked bar plots describe the distribution of indicated literature susceptibility categories across indicated aggregate similarity score quantiles.

ACE2 sequences classified by MrSARS as susceptible with high confidence captured at least 80% of putatively susceptible species in each examined dataset (Table S4). Medium confidence sequences fell into susceptible, ambiguous, and resistant categories across all examined study types, though most MrSARS medium confidence ACE2 sequences found counterparts in ambiguous or resistant categories (Fig. 4a). Apart from Fischhoff et al., MrSARS AS scores tended to roughly track with animal susceptibility reports across the distinct literature datasets (Fig. 4b).

MrSARS was developed to establish a ranking of sequence susceptibility to infection, which can enable researchers to prioritize species with higher receptor sequence rankings for experimental validation. We compared MrSARS AS score quantiles to the datasets in our meta-analysis (Fig. 4c). More than 90% of AS scores falling in the top 5% were identified as susceptible species across in silico, in vitro, Damas et al., and Fischhoff et al. datasets. At least 75% of species reported as susceptible by in silico and in vitro studies are represented in the top 3 deciles of MrSARS AS scores. Species with ambiguous infection susceptibility are predominantly represented in middle and lower quantiles of in silico and in vitro studies respectively. Putatively resistant species generally occupied lower AS score quantiles at a higher proportion across in silico, in vitro, Damas et al. and Fischhoff et al. datasets. The proportion of putatively resistant species tended to be higher in upper AS score quantiles in the Damas and Fischhoff datasets. In cellus and in vivo datasets did not show a clear correlation with MrSARS AS scores. In summary, MrSARS correlated with previous in silico and in vitro studies while adding predictions for species not previously examined.

## Discussion

In this study, we developed MrSARS, a receptor sequence similarity analysis pipeline intended to determine what species may be susceptible to a virus that utilizes a chosen receptor protein. MrSARS takes an input of virus entry protein targeted receptor residues, and a flexible number of reference sequences to generate an AS score that can be used to rank species according to their predicted similarity to hosts with known virus-receptor binding interfaces. Using the SARS-CoV and SARS-CoV-2 receptor ACE2 as a model, we evaluated AS score validity by bootstrapping MrSARS with randomly sampled reference sequences to generate a random AS score distribution for each query sequence. This analysis allowed us to categorize our AS scores as susceptible with high confidence, medium confidence or putatively resistant to SARS-CoV-related sarbecovirus infection according to their probability of diverging from this random distribution. Our experimental data were generally consistent with MrSARS rankings and confidence categorizations, and previously reported in silico and in vitro reports.

MrSARS differs from existing susceptibility prediction tools in several ways. MrSARS can be used with any receptor protein for which a receptor-virus entry protein interaction is known. MrSARS is flexible and can be executed with any number of chosen reference sequences. MrSARS can be applied to the analysis of host susceptibility to a single virus strain or a clade of viruses that utilize the same binding interface. Thus, susceptibility predictions need not be limited to similarity to a single chosen reference species; phylogenetically diverse hosts can be included in the analysis. Its output is a straightforward ranking that is easily interpretable and allows the researcher to prioritize relevant species for experimental testing and virus surveillance. MrSARS is not computationally expensive and can be executed on a personal computer.

The high number of susceptible species identified by MrSARS (271/448, 60.5%) is consistent with existing studies that have applied various computational methods to infer animal susceptibility to SARS-CoV-2^23,44,45^. This suggests that SARS-CoV-2 and related sarbecoviruses are particularly promiscuous in terms of their ability to access new species. Similar analyses of other virus receptors that are composed of single proteins like MERS receptor DPP4^22^, RD-114 retrovirus receptor ASCT2^46^ or Seneca valley virus receptor ANTXR1^47^ may determine whether such a wide spectrum of possible target species is specific to sarbecoviruses or extends to other virus lineages.

It is notable that Artiodactyla appears to be a diverse animal clade primed for sarbecovirus invasion. White tailed deer have become an established reservoir, and evolutionary hotbed of SARS-CoV-2^31,48–52^. MrSARS and other SARS-CoV-2 spillover prediction studies have suggested that other artiodactyl lineages may be prime targets for SARS-CoV-2 spillover. Whale and dolphin AS scores fall in the top 10% of all analyzed species (Table 1), which is consistent with published studies looking at ACE2 similarity, spike-ACE2 binding, pseudovirus entry and animal proximity to humans and human wastewater^23,53–56^. The AS score of reindeer is the second highest in our dataset, suggesting these animals, which are widely farmed in northern latitudes, could be susceptible to SARS-CoV-2.

SARS-CoV-related sarbecoviruses have evolved to utilize a deeply conserved and functionally constrained receptor in ACE2. This purifying selective pressure is evident in the high degree of sequence conservation within the RBDs of SARS-CoV and SARS-CoV-2 lineages (Fig. S12). Minor changes in the spike receptor binding domain, such as at position 501 for SARS-CoV-2, may permit their spread to new hosts that only have minor differences at spike-bound residues of ACE2. The high number and diversity of sarbecoviruses circulating in bats (Fig. 3a) has resulted in the sarbecovirus-induced evolutionary diversification of ACE2 alleles in the bat lineage^22,23^ and within individual horseshoe bat species^24,29^. This may explain why most bat sequences fall within the medium confidence susceptibility category in our MrSARS analysis.

The discrepancy in animal susceptibility across study types (Figs. 4, S8-S11) may be attributable to several factors. On a technical level, some reports generated data that is not amenable to binary classification. Frequently, data are presented without a clear quantitative distinction, which is prevalent among sequence analysis studies that fall into the in silico class. In vitro and *in cellus* experiments generate data that is distributed on a spectrum of values.

Virus strain is likely to be another major contributor to conflicting susceptibility reports because species may be reported as susceptible to one virus strain but resistant to another. If distinct strains are used in different studies to test animal susceptibility, this discrepancy will be captured in our literature analysis. This issue becomes particularly apparent when examining in vitro and in cellus studies (Figs. S10, S11). The literature associated with SARS-CoV, SARS-CoV-2, and related sarbecoviruses examines a broad swath of viruses, which results in diverse ACE2 receptor usage outcomes (Figs. S10, S11). Our data suggest that SARS-CoV-2 variants can have distinct host receptor preferences (Fig. 2d–h). For example, mouse ACE2 cannot be used by ancestral or delta variant SARS-CoV-2; but other variants can utilize mouse ACE2 (Fig. 2d–h)^36,57,58^. These examples illustrate how small but significant differences in spike RBDs can dramatically influence sarbecovirus tropism.

Considering that the majority of ACE2-utilizing sarbecoviruses have been isolated from bats^32^, it is somewhat surprising that a majority of bat sequences fall within our medium confidence category (Fig. 1f). This discrepancy could be explained by the hypothesis that persistent circulation of sarbecoviruses in bats has likely driven ACE2 allele diversification as a means of evading sarbecovirus infection. This hypothesis is consistent with the literature showing a high level of ACE2 allelic variation within individual bat species^24,25^, and significant positive selection across the bat lineage^22,23^. It is possible that few bat ACE2 sequences fall into our high confidence category because some sarbecoviruses bind ACE2 in a way that is comparable to SARS-CoV and SARS-CoV-2. Since our analysis with MrSARS utilizes reference ACE2 sequences known to bind SARS-CoV-2, more dissimilar bat ACE2 sequences putatively targeted by other sarbecoviruses would be expected to fall down our rankings. Thus, our pipeline can distinguish between ACE2 sequences relevant to SARS-CoV-2 and SARS-CoV susceptibility and those that are not.

Receptor sequence analysis with multiple reference species can be a useful tool to identify animals at risk for spillover infections. As illustrated in our meta-analysis (Fig. 4), susceptibility predictions generated by any computational tool, including MrSARS, should not be interpreted as authoritative or comprehensive. The significant number of contradictory animal susceptibility reports across experimental studies (in vitro and in cellus) highlights the challenge of assessing virus spillover potential in the absence of in vivo studies. While receptor-dependent virus entry represents a necessary first step in a viral infection, successful cell entry is not sufficient to accurately assess a spillover risk. Several ecological and immunological factors are similarly critical in determining the likelihood of a spillover event^59^. (i) There must be sufficient overlap in animal habitats that provide a virus the opportunity to infect another host. If, for example, whales and dolphins do not consistently interact with humans, then it is unlikely that SARS-CoV-2 can make the leap into these species. (ii) The virus must have a mode of transmission that is conducive to invading another species. Because SARS-CoV-2 is largely transmitted by aerosols, it is apparent how this virus was able to move from bats through a putative intermediate host into humans and on to other animals like deer, dogs, and cats. However, it is not clear if SARS-CoV-2 viral particles are stable in aquatic and marine environments. Thus, while marine mammals may be susceptible at the receptor level, they may never be sufficiently exposed to SARS-CoV-2 to contract this virus. (iii) The receptor must be expressed at sufficient levels in tissues that will encounter invading virus particles. A respiratory virus cannot infect respiratory epithelial cells if the receptor protein is not present in these tissues. (iv) Virus entry represents the first step of an infection. If a virus is incompatible with host cellular machinery or incapable of overcoming innate and adaptive immune responses, then the virus will not establish a foothold in its new host or be transmitted between individuals^19^. This virus behavior has been observed for several animals where evidence of SARS-CoV-2 infection has been detected but onward transmission has not^60–64^. Tools like MrSARS should be utilized as an initial screening tool to identify candidate species for experimental validation and subsequent surveillance in the field. It is incumbent on researchers to consider additional ecological and physiological factors that may contribute to spillover risk. In the future it may be valuable for computational susceptibility predictions to be integrated into ecological modeling and virology studies to generate a comprehensive assessment of virus spillover risks.

## Materials and methods

### Cell line

293T cells were purchased from ATCC (CRL-3216) and cultured in Dulbecco’s Modified Eagle Medium (DMEM, Gibco 11965092) supplemented with 10% heat-inactivated fetal bovine serum (FBS, Gibco 10082147), 1% antibiotic–antimycotic solution (Gibco 15240062), and 1 mM sodium pyruvate (Gibco 15240062).

### Plasmids and antibodies

All non-human, species-specific ACE2- and GFP- sequences used for pseudovirus infection assays in this study were cloned into the pCMV6-humanACE2-MYC-FLAG (OriGene, RC208442) overexpression plasmid. Reference ACE2 sequences for mouse (NM_001130513.1), American mink (XM_044236017.1), pig (NM_001123070.1), narwhal (XM_029239971.1), Coquerel’s sifaka (XM_012638731.1), small Madagascar hedgehog (XM_004709945.2), Himalayan frog (XM_018563056.1), ruff (XM_014960219.1), sinicus bat (MT394185.1, KC881004.1), and affinis bat (MT394203.1) were downloaded from the NCBI nucleotide database. Reference ACE2 sequences for reindeer and white-tailed were not available in the NCBI nucleotide or gene databases. Thus, the narwhal ACE2 nucleotide sequence was used as a query for a BLASTn search of the reindeer genome assembly (GCA_902712895.1). Matching reindeer sequence fragments were downloaded, and a multiple sequence alignment was generated with MAFFT (implemented within Jalview^65^) using narwhal ACE2 as a reference. These sequence fragments were then stitched together using the Jalview consensus sequence output. ORF integrity was assessed with ORFfinder^66^. ORF disrupting nucleotides were manually corrected using the reindeer genome assembly as a reference where possible. If further ORF-disrupting sequence differences remained, narwhal ACE2 sequence was used as a secondary reference. The same sequence assembly process was executed for white-tailed deer (GCF_002102435.1) using newly identified reindeer ACE2 as the reference. All ACE2 ORF-encoding nucleotide sequences were codon optimized for expression in human cells using the IDT codon optimization tool. The GFP ORF was PCR amplified from the pEGFP-N1 plasmid (provided by Dr. Medzhitov, Yale University). Codon-optimized ACE2 ORF sequences were synthesized as gene fragments (mouse, pig, narwhal, reindeer, Coquerel’s sifaka, sinicus bat allele 1443, affinis bat), which contain 5’ KpnI and 3’ MluI restriction sites for downstream cloning into above described the pCMV6-humanACE2-MYC-FLAG backbone, or directly synthesized as complete expression plasmids (hedgehog, ruff, Himalayan frog, sinicus bat allele 3358) by Twist Bioscience. ACE2 gene fragments were cloned into KpnI (NEB R3142S) and MluI (R3198S) double-digested pCMV6-MYC-FLAG plasmid backbone using the InFusion cloning kit (Takara 638,948). pCDNA3-SARS-CoV-2-beta-S (QRN78347.1) expression plasmid was provided by Dr. Wilen (Yale University). pCAGGS-SARS-WIV1-S (KF367457.1), pCAGGS-SARS-CoV-Tor2-S (YP_009825051.1), pCAGGS -SARS-CoV-2-WA1-S (QIQ50192.1), pCAGGS-SARS-CoV-2-delta-S (UBG84005.1), pCAGGS- BANAL236-S (MZ937003), and pCAGGS-BANAL52-S (MZ937000) were synthesized as gene blocks by Twist Bioscience and subcloned into XbaI (NEB R0145S) and NotI (NEB R3189S) double-digested pCAGGS plasmids by Gibson Assembly (NEB HiFi M5520). pTwist-SARS-CoV-2-BA1-S (UFO69279.1), pTwist-SARS-CoV-2-BA4-S (UPP14409.1), expression plasmids were directly synthesized by Twist Bioscience.

All antibodies used in this study are commercially available. Anti-MYC-HRP (71D10, #14038) and anti-GAPDH-HRP (14C10, #3683) were purchased from Cell Signaling Technology.

### Western blot

Whole cell lysates from cultured 293T cells cultured in a 24-well format were prepared using 100 µL RIPA buffer and supplemented with complete protease inhibitors (Roche 11697498001) following manufacturers specifications. Lysates were agitated for one hour at 4 °C and clarified by centrifugation at 13,000xg for 15 min. Lysate protein concentration was determined by DC protein assay (BioRad 5000111) as outlined by the manufacturer. One quarter volume 5 × Laemli buffer was added to each sample and heated at 95 °C for five minutes. Approximately 30ug of protein was then separated by SDS-PAGE (BioRad 4569034), transferred to methanol pre-treated PVDF membrane (Millipore IPVH00010). Membranes were washed with TBST, blocked with TBST containing 5% milk, and incubated overnight at 4 °C in appropriate HRP-conjugated primary antibodies.

### Angiotensin conversion assay

Angiotensin conversion assays were performed according to the manufacturers (Abcam ab273297) protocol. Briefly, ACE2- or GFP-transfected 293T cells were harvested by trypsinization and lysed using 400 µL of lysis buffer. Lysates were clarified by centrifugation at 16,000xg for 10 min at 4 °C. Protein concentrations were quantified using a reducing agent-compatible kit (ThermoFisher R33200) following the manufacturer’s protocol. The angiotensin conversion assay was performed in triplicate with 2 µL of each cell lysate in the presence or absence of an ACE2 inhibitor included in the kit. Fluorescence was measured kinetically and at room temperature with a cytation5 plate reader over 30 min.

### Sequence similarity analysis

We obtained vertebrate ACE2 protein coding sequences from several NCBI databases. Reference ACE2 coding sequences were manually downloaded from the NCBI RefSeq database^67^. Using EDirect^20^, we searched the NCBI nucleotide database^21^ to obtain a list of unique ACE2 accession identifiers and downloaded the corresponding GenBank entries. Additional Rhinolophus and Hipposideros bat ACE2 GenBank entries were downloaded with EDirect using a list of nucleotide accessions reported by Dr. Frank^22^. We then wrote a custom Biopython script^68^ to extract peptide sequences from these GenBank entries and generate a protein coding sequence multifasta file with headers formatted as ‘accession_species_name’. Several Rhinolophus bat species, Rangifer tarandus, and Oncorhynchus mykiss sequences, which were missing from our initial EDirect search, were manually assembled from genome assemblies (Rangifer tarandus), or manually downloaded from NCBI ortholog or nucleotide databases (Rhinolophus bat species and Oncorhynchus mykiss). The resulting nucleotide sequences were then translated into peptide sequences with transeq^69^ and saved as a multifasta file with the same header format. RefSeq, GenBank, bat and manually assembled ACE2 peptide sequence multifasta files were concatenated into a single multifasta file, which was used in downstream file processing and sequence analysis processes. This multifasta file was processed with a custom Biopython script to ensure only full length ACE2 sequences with unique accessions were retained. The resulting multifasta file was iteratively passed through MAFFT^70^ and MUSCLE^71^ sequence alignment programs to generate sequence alignments. The alignment was manually inspected for non-ACE2 sequences, which were then removed. This process was repeated until only high quality ACE2 sequences were retained, and a high-quality sequence alignment was obtained. The resulting alignment was manually refined, and spike-binding residues (as defined by^23^) were extracted using a custom Biopython script. The Damas 2020 spike-bound ACE2 residue dataset was subsequently appended to our multifasta file. Duplicate accessions and species-specific sequences were removed to reduce redundancy in downstream similarity analyses. This resulted in a final spike-bound ACE2 peptide sequence dataset that was used in our downstream sequence similarity analyses with MrSARS.

MrSARS is a flexible, python-based program that principally calculates sequence similarity scores between given sequences and a set of predefined reference sequences. MrSARS takes an input multifasta file and a list of reference sequence headers, which must be present in the input multifasta file. Our program utilizes Biopython’s SeqIO and PairwiseAligner modules to generate pairwise alignments between every ordered pair of sequences using the BLOSUM62 substitution matrix. For every sequence provided in the input multifasta, MrSARS creates a ‘Species’ object containing the following: a query sequence, a self-similarity score (i.e., the BLOSUM62 score resulting from comparing the query sequence to itself), and a dictionary of similarity scores comparing the query sequence to every other sequence. These scores are then normalized by dividing by the self-similarity score. An aggregate similarity (AS) score for each sequence is then calculated by summing the normalized similarity scores from each given reference species. Finally, an output comma-separated values (CSV) file is generated that contains reference-specific normalized similarity scores and aggregate similarity scores for each sequence. MrSARS can be executed in the python command line or using a graphical user interface implemented with the Tkinter package.

We additionally developed a modified version of MrSARS (MrSARS-sampler) that runs the same logic as MrSARS, but with 1000 sets of 5 randomly sampled references from the input multifasta. The program then provides an output CSV file where every column represents a species, with each row storing the AS scores against one set of randomly sampled references. Finally, the last row stores the AS scores against the provided reference species. Species names were extracted from the MrSARS output, their format adjusted for NCBI compatibility, and appended to the table as a new column with a custom python script.

To define confidence categories for our ‘true’ reference AS scores, the output of MrSARS-sampler was subsetted into two groups; one containing randomly generated aggregate similarity scores (see above) and the other containing scores generated using the input reference sequence file. Percentiles for each ‘true’ reference AS score were calculated by applying the empirical cumulative distribution function (ecdf implemented in R) to the scores, which ranked the reference scores within the context of the sampled scores. These percentiles were then visualized as a histogram (see Fig. 1c). After categorizing percentiles into bins of ‘high’, ‘medium’, and ‘resistant’, a scatter plot was generated and colored according to these categories.

We obtained animal taxonomy information from the NCBI taxonomy database for species encoding an ACE2 sequence analyzed in this study. We wrote a custom bash script that utilizes EDirect to search for and extract species-associated taxonomic classifications ranging from family to genus. The resulting data was saved as a tab-delimited file and subsequently manually processed to ensure taxonomic information was appropriately classified. This taxonomic data was then joined^72^ with the MrSARS output file based on species names contained in both data frames and duplicate rows were removed by retaining only unique sequence identifiers (‘Record_ID’). This data frame was used to quantify what animal orders compose our ACE2 sequence dataset (Fig. 1) and subset species in our literature analysis (Fig. 4).

### Pseudovirus production

To produce sarbecovirus spike pseudotyped VSVpp-Rluc viral particles, 293T cells were seeded in 10 cm dishes and allowed to adhere overnight. Cells were then transfected with 8 µg of sarbecovirus spike overexpression plasmid using Lipofectamine 3000 (ThermoFisher L3000008) according to manufacturer’s instructions. Cell culture media was replaced six hours post-transfection. At 20 h post-transfection, culture media was replaced with 5 ml prewarmed media containing 50 µL of VSVpp-Rluc-VSVg virus and incubated at 37 °C for one hour. Cells were then washed 3 × with prewarmed PBS to remove unbound inoculum. Then fresh media, supplemented with anti-VSVg antibody (Millipore 8G5F11) diluted to a final concentration of 0.1 ug/mL, was added to washed cells. Virus-containing supernatants were harvested 24 h post-infection, centrifuged at 3000 rpm for 10 min at 4 °C to remove cell debris, and the virus was aliquoted and stored at −80 °C.

### Infection assays

Approximately 3 × 10^5^ 293T cells were transfected in a 96-well format with MYC-FLAG-tagged GFP or ACE2 overexpression constructs using Lipofectamine 3000 (ThermoFisher L3000008). Following a 24 h incubation period, cells were infected with unconcentrated, spike protein pseudotyped VSVpp-Rluc reporter viruses diluted in DMEM lacking FBS and phenol red. Pseudoviruses were diluted at a virus to media ratio ranging from 1:5 to 1:100. Virus dilutions were empirically determined to generate a luciferase signal that was significantly above background and low enough to accurately measure differences in luciferase signal. After a 24 h incubation period, cells were lysed, and luciferase activity was measured with the Renilla Luciferase Assay System (Promega E2820) according to the manufacturer’s instructions. Luciferase activity was measured using a BioTek Cytation 5 plate reader. Pseudovirus luciferase signal for ACE2-MYC-FLAG expressing cells was normalized to signal generated in GFP-MYC-FLAG expressing cells.

### Protein structure analysis

Crystal structures for PDB entries 2AJF^73^ and 6MOJ^40^ were processed and images with (Fig. 3b, c) with ChimeraX^74^ which was developed by the Resource for Biocomputing, Visualization, and Informatics at the University of California, San Francisco, with support from National Institutes of Health R01-GM129325 and the Office of Cyber Infrastructure and Computational Biology, National Institute of Allergy and Infectious Diseases.

### Sarbecovirus spike sequence alignment phylogenetic tree construction

For Fig. 3a, we assembled a list of putatively ACE2-utilizing sarbecovirus accession IDs using virus references described by Drs. Wells, Seifert, and Chan^32,75,76^. Sequences referenced in these publications were obtained from the NCBI nucleotide database^21^. This was achieved by using AnnotationBustr^77^ to directly download sarbecovirus spike sequences, and by manually downloading spike sequences for nonconforming NCBI accessions. Following this search, all identified sequences were concatenated into a master multifasta file (supplemental data). Duplicated accessions were manually removed from this multifasta file. These spike-encoding nucleotide sequences were converted to an amino acid multiple sequence alignment with Jalview^65^ and aligned with MAFFT^70^ using default settings. This multiple sequence alignment was passed through IQ-TREE^78^ to generate a newick-format phylogenetic tree, which was converted into a graphical tree with FigTree v1.4.4.

For the sequence alignment shown in Fig. S12, indicated amino acid sequences were aligned with MAFFT using default settings. Residues shown in Fig. S12 were manually extracted using the JalView graphical interface and colored according to the default BLOSUM62 similarity matrix that is implemented in Jalview.

### Literature meta-analysis

Our literature search for animal susceptibility to SARS-CoV/SARS-CoV-2 and related sarbecoviruses encompasses peer-reviewed and pre-print articles spanning 2003 to 2024. The complete record of this search (including article metadata, virus information, analyzed host species, and data type) is encompassed in Table S1. Virus susceptibility for a given species was classified in a binary format where species resistance was assigned 0 and species susceptibility was assigned 1. The absence of data for a given study type is denoted by NA. This table served as the literature data source for the following analyses.

We quantified the intersection of species analyzed in the literature from various experimental and computational approaches (Table S1) with the similarity predictions generated by MrSARS (Table S5) and utilized the R package ggVennDiagram^79^ to generate an upset plot (Fig. S7).

Before conducting further data processing, Fischhoff et al. and Damas et al. data were each separated from Table S1. Fischoff, Damas, and the broader literature data were then processed as follows. The literature data (Table S1) was reformatted into ordinal values to allow for direct comparison of reported species susceptibility with MrSARS predictions. We first tallied the number of studies reporting susceptibility and resistance for each species across all study types (in vivo, in cellus, in vitro, and in silico). This produced a summary table containing species-specific counts of susceptible versus resistant outcomes across all four across modalities. Species were classified as (i) susceptible if at least one study reported susceptibility and no studies reported resistance, (ii) resistant if at least one study reported resistance and no studies reported susceptibility, and (iii) ambiguous when both susceptibility and resistance were reported for the same species. Damas et al. bin species into five susceptibility categories ranging from very high to very low susceptibility (Fig. 4b). To enable direct comparison of the Damas study with MrSARS, species categorized as very highly and highly susceptible were binned into one susceptible category. Species with very low and low predicted susceptibility were binned into a putatively resistant category (Fig. 4a). Species defined by Damas as having a medium likelihood of infection susceptibility were considered comparable to MrSARS medium confidence category.

To enable direct comparison between MrSARS and our literature datasets, categorical susceptibility assignments were converted into ordinal numeric values. Susceptible, resistant and ambiguous species were assigned a value of 1, 0, and 0.5 respectively. Identical ordinal susceptibility scores were assigned to MrSARS percentiles (Table S2). Sequences in the 95th percentile (classified in sequence similarity analysis as high confidence) were assigned a value of 1, while sequences in the 80th–95th and below the 80th percentile were designated as having moderate susceptibility and resistant, and assigned a value of 0.5 and 0 respectively. The literature and MrSARS datasets were then intersected according to common species.

The performance of MrSARS compared to the above-described study types and studies was visualized with using the following approaches. Connected scatter plots for each indicated study type and dataset were independently generated to visually assess MrSARS’ species-specific susceptibility predictions vs the indicated study type. Scatter plots mapping MrSARS AS score and percentiles were generated and the data points colored according to study type categories. Stacked bar plots were generated to plot AS score quantiles and describe the proportion of susceptible, ambiguous and resistant species encompassed in each quantile. The R package ComplexHeatmap was then used to create heatmaps for each major mammalian group and model organisms (see Fig. S9).

To assess the degree of conflicting data on animal susceptibility (see Figs. S10 and S11), we used the following approach. First, we calculated the number of times each species was reported as susceptible or resistant across in silico, in vitro, in cellus, or in vivo studies. Because we were interested in assessing the degree of conflicting reports, we removed species with complete consensus across all study types. This filtering step retained species for which at least one study type contained conflicting reports. We then applied a square root transformation to the product of opposing counts (positive and negative) to prepare the data for visualization. This transformation aims to provide a balanced view of the data, highlighting areas with significant discrepancies. We then used heatmaps to visualize this transformed data. We also generated dot plots using ggplot2 to depict the number of positive and negative findings for each species and associated study type.

### Statistical analyses

Least squares regression and Dunnett’s post-hoc pairwise comparison tests, used to assess differences in pseudovirus infection rates relative to GFP and human ACE2 controls, were executed in R using the emmeans package^80^.

To perform Cohen’s κ tests, 3 × 3 contingency tables summarizing the joint susceptible, ambiguous and resistant classifications of MrSARS and each literature study type were first constructed (Table S4). The vcd package^81^ was then used to perform a weighted Cohen’s κ test to account for the ordered nature of the susceptibility categories and to penalize disagreements in proportion to their ordinal distance. Krippendorf’s α calculations were executed using the irr package^82^. The ordinal susceptibility classifications defined in the MrSARS and literature data were encoded numerically and a method x species matrix was assembled. Krippendorf’s alpha was then calculated using this matrix. Krippendorff’s α uncertainty was estimated non-parametrically via bootstrap resampling. A 95% confidence interval was determined by resampling species with replacement 5,000 × and calculating α for each bootstrap sample.

### Computational tool usage

MrSARS/-sampler were implemented in Python 3.9.18, biopython 1.85, and pandas 2.3.1. All data processing and statistical analyses were conducted with R version 4.5.1 (2025-06-13). Experimental design images (Fig. 2a) were prepared with BioRender.

## Supplementary Information

Below is the link to the electronic supplementary material.

Supplementary Material 1

Supplementary Material 2

Supplementary Material 3

Supplementary Material 4

Supplementary Material 5

Supplementary Material 6

Supplementary Material 7

## Data Availability

MrSARS/-sampler code and code used for database mining, sequence processing, data processing and plotting, and statistical analysis have been uploaded to github: https://github.com/jofrank1988/MrSARS. Materials generated for this study (cell lines, plasmids, and primers) are available upon request from J.A. Frank or A. Iwasaki. Raw experimental data will be provided by J.A. Frank upon request.
