# Supplementary material for: Systematic multi-reference vertebrate ACE2 sequence similarity analysis predicts species susceptibility to SARS-related sarbecoviruses
Source: Sci Rep. 2026 Mar 18;16:13995. doi: 10.1038/s41598-026-41410-9 (PMC13133125; doi:10.1038/s41598-026-41410-9)
Supplement: Supplementary file 1 — Supplementary Material 1 [file 41598_2026_41410_MOESM1_ESM.pdf]

Raw ACE2 (anti-MYC-HRP) Western Blot of ACE2-tranfected 293T cells

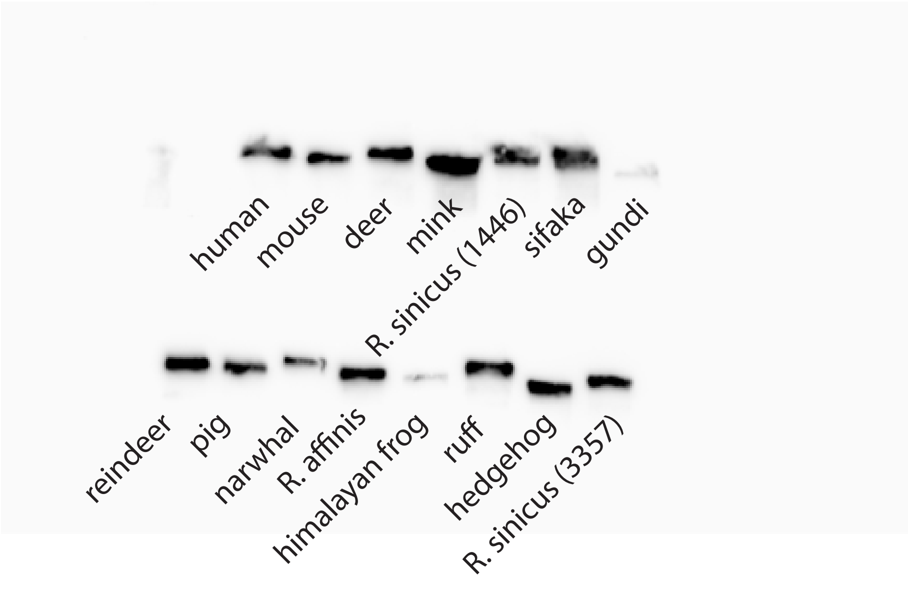

Raw GAPDH (anti-GAPDH-HRP) Western Blot of ACE2-transfected 293T cells.

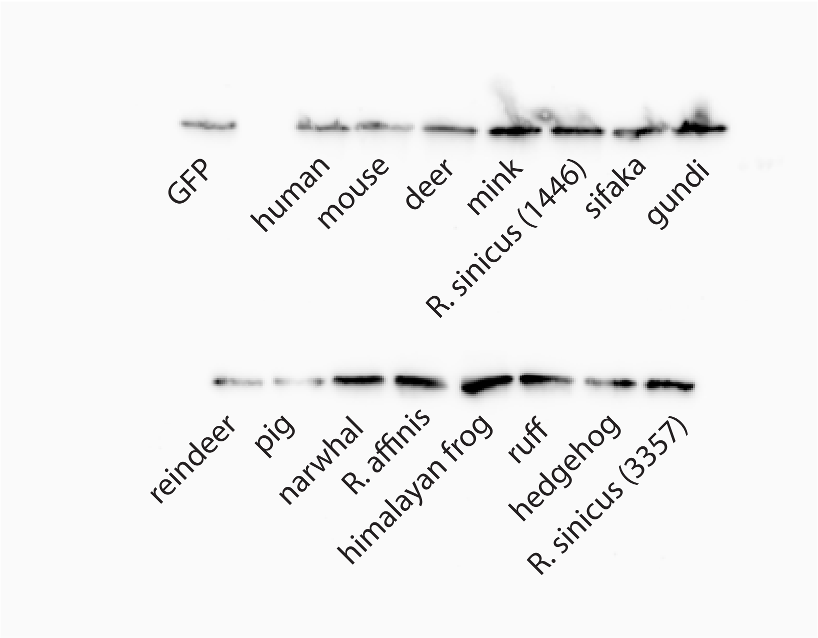

Above images are directly taken from BioRad ChemiDoc Imager captured in luminescence automatic acquision mode. Blots were cut to enable concurrent imaging of all ACE2-MYC-FLAG and GAPDH bands. Left-most band in GAPDH is representative of a sample expressing GFP-MYC-FLAG. The GFP band is not visible in the ACE2 blots because GFP-MYC-FLAG migrates at 30.55kDa, which would place it in the size range of GAPDH.
