# Supplementary material for: Systematic multi-reference vertebrate ACE2 sequence similarity analysis predicts species susceptibility to SARS-related sarbecoviruses
Source: Sci Rep. 2026 Mar 18;16:13995. doi: 10.1038/s41598-026-41410-9 (PMC13133125; doi:10.1038/s41598-026-41410-9)
Supplement: Supplementary file 2 — Supplementary Material 2 [file 41598_2026_41410_MOESM2_ESM.pdf]

Supplemental Figures:

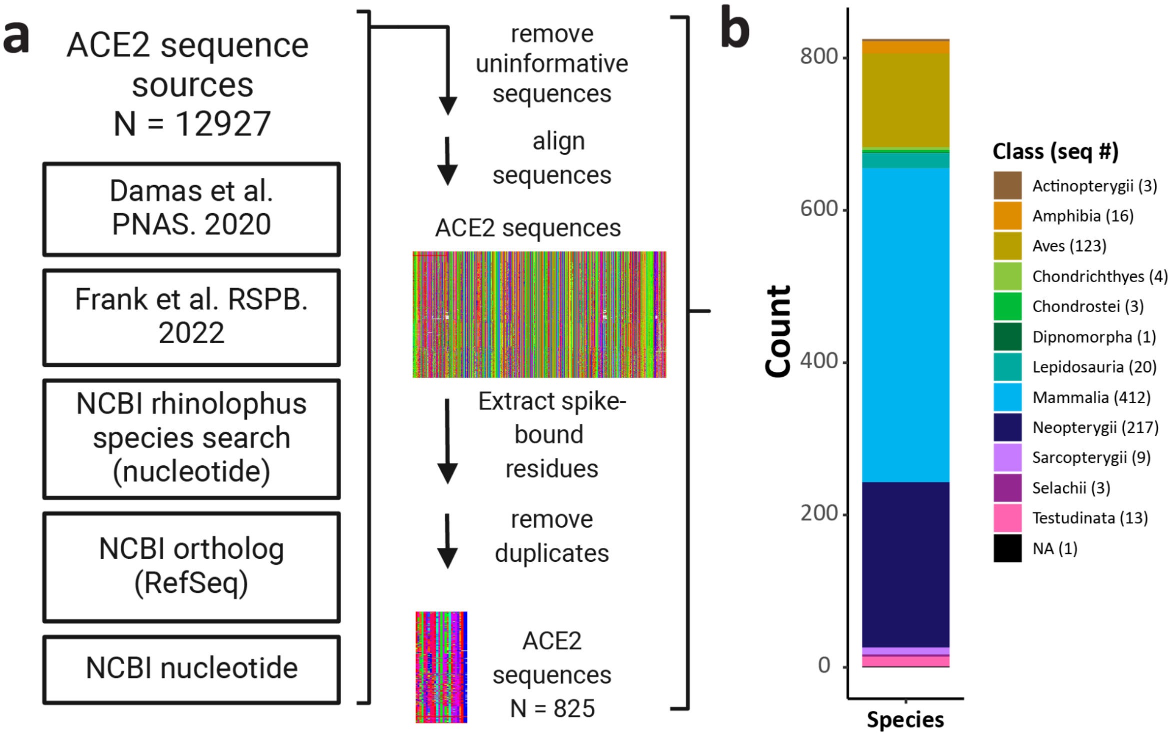

Figure S1: ACE2 peptide sequence dataset assembly and processing. a) ACE2 sequences were assembled from indicated sources. Non-ACE2 or incomplete sequences were removed from the input dataset. Remaining sequences were aligned with MAFFT, and spike bound residues (defined by Damas et al. 2020) were extracted. Duplicate sequences from each species were removed leaving only spike-bound ACE2 sequences that are unique for each species. **b)** Stacked bar plot illustrates the indicated number of ACE2 sequences represented in each animal class.

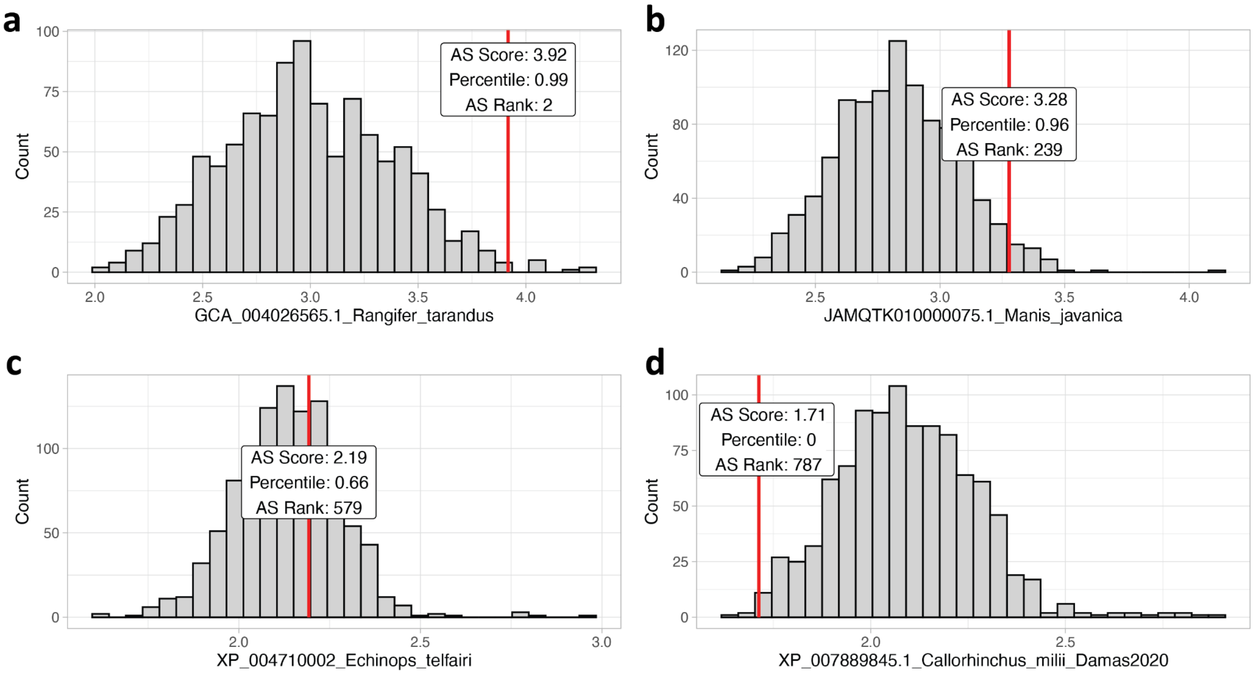

Figure S2: Comparison of true MrSARS aggregate similarity scores vs a distribution bootstrapped random reference aggregate scores. a-d) True aggregate similarity (AS) scores for reindeer (a), pangolin (b), hedgehog (c), and ghost-shark (d) are plotted against a distribution of AS scores generated by randomly sampled reference species. Histograms represent the counts of AS scores generated by randomly sampled reference species and assigned to indicated bins. Red vertical lines denote the AS score generated by MrSARS using true reference species. Overlayed boxes present AS score, percentile within the random AS score distribution, and AS score-based ranking.

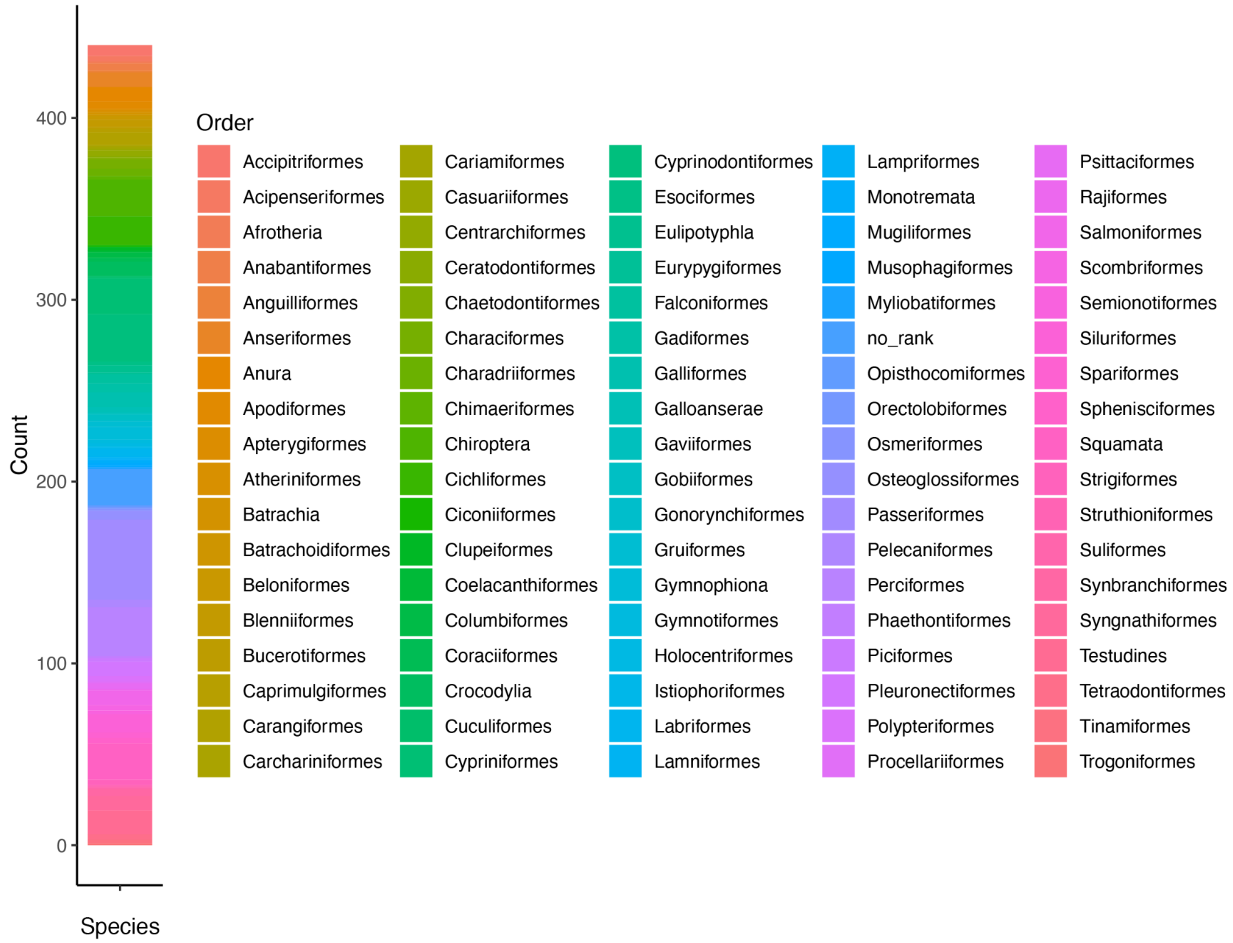

Figure S3: MrSARS ACE2 analysis identifies putatively resistant species. a) Vertebrate animal order counts of ACE2 sequences categorized as putatively resistant based on MrSARS analysis.

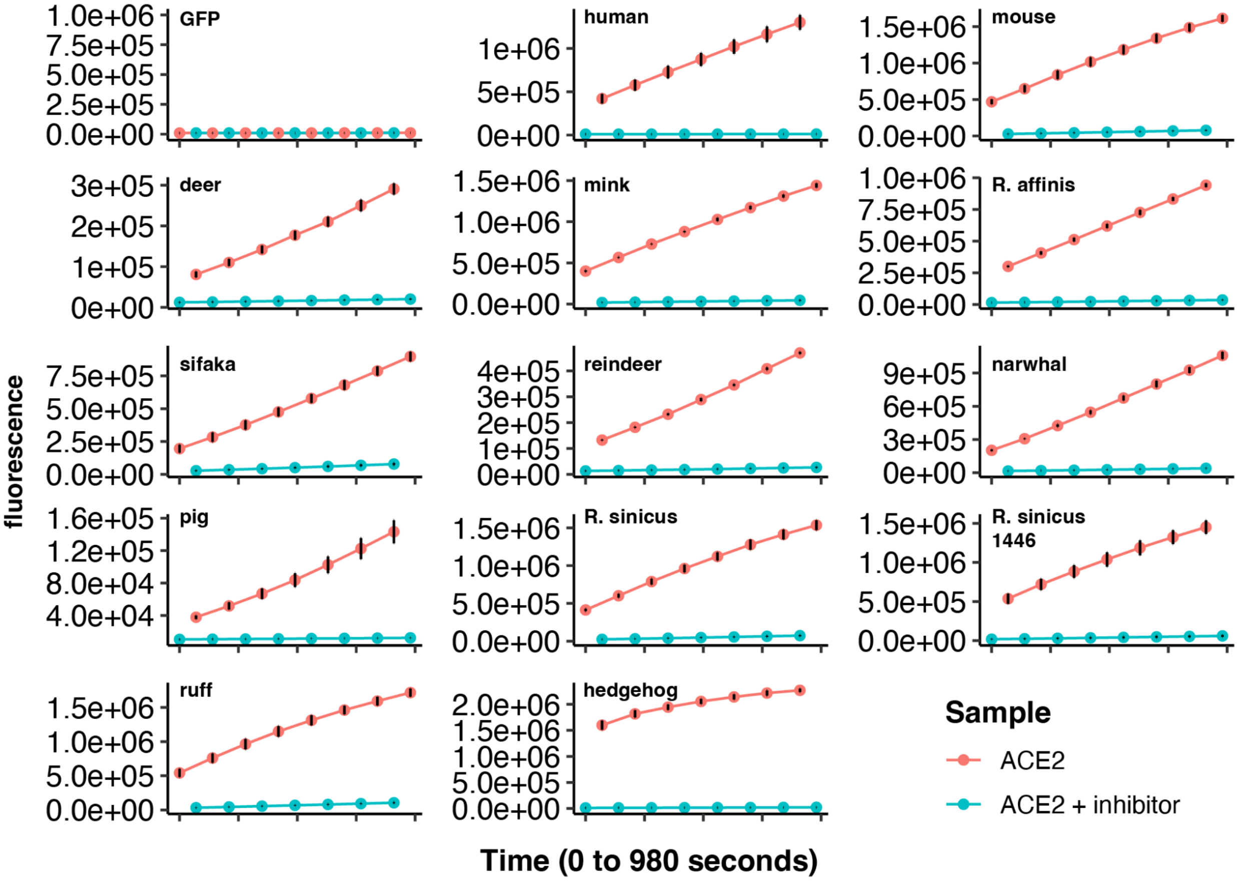

Figure S4: Vertebrate ACE2 expression plasmids produce enzymatically functional protein. Angiotensin conversion assays executed with cell lysates generated from 293T cells transiently transfected with pCMV6 expression constructs encoding ACE2 from indicated species. Red lines denote assays executed with ACE2-containing samples. Blue lines denote assays executed with ACE2-containing samples and added ACE2 inhibitor. Line plots encompass 3 technical replicates. This figure is representative of 3 independent experiments.

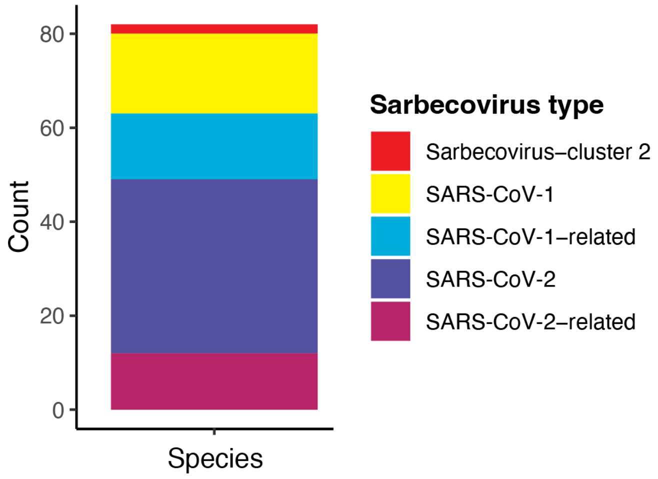

Figure S5: Count of unique sarbecovirus strains included in literature analysis shown in Figure 4. Stacked bar plot presents the number of unique viruses that fall within the indicated sarbecovirus categories.

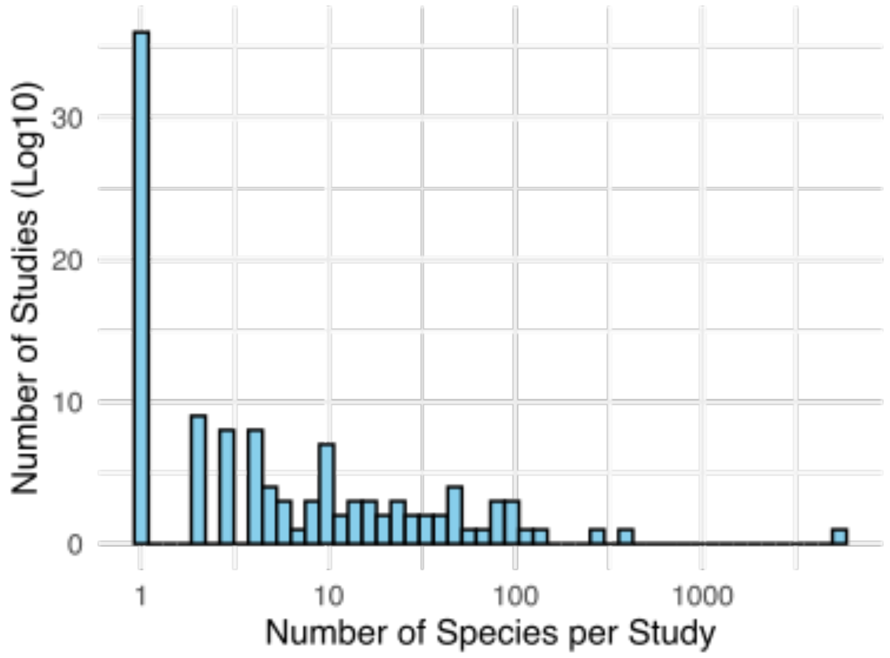

Figure S6: Summary of number of species examined per published study. Histogram represents the distribution of articles examining one or more species per study.

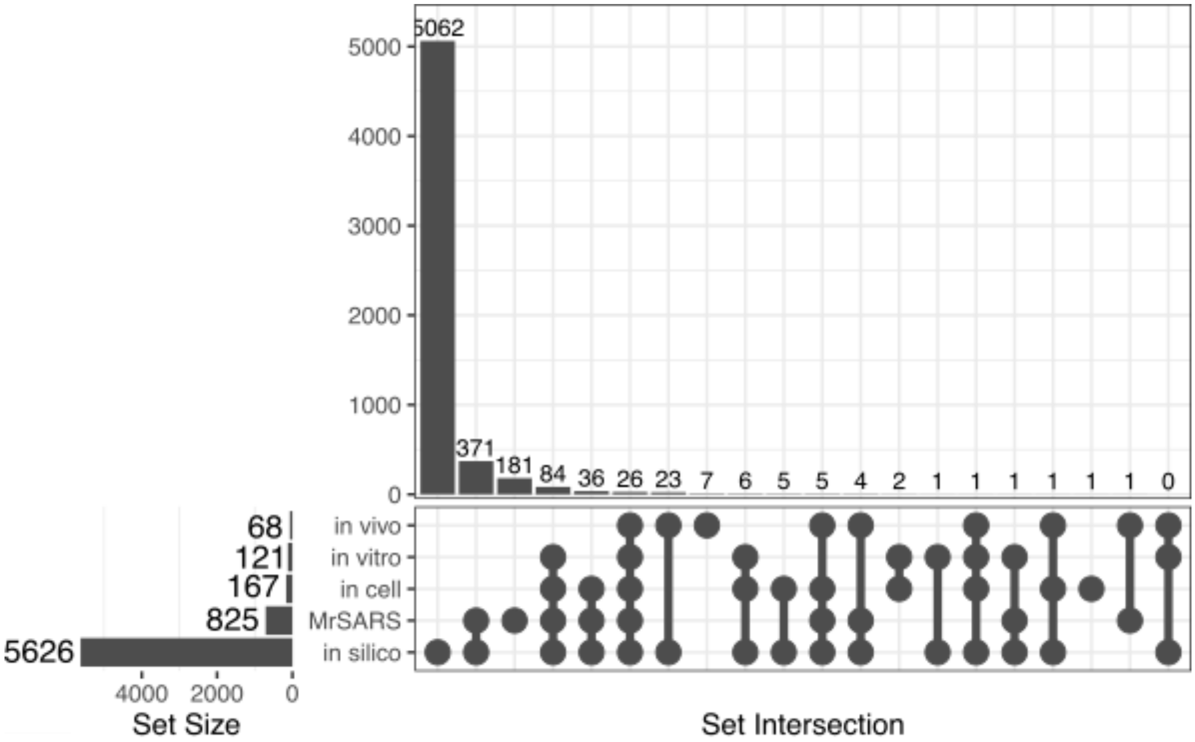

Figure S7: Upset plot summarizing the overlap between species examined by MrSARS and published studies. The Unique species number represented in each study type and sequences examined by MrSARS is summarized in left horizontal bar plots. Vertical bar plots represent the number of species that overlap between indicated study types.

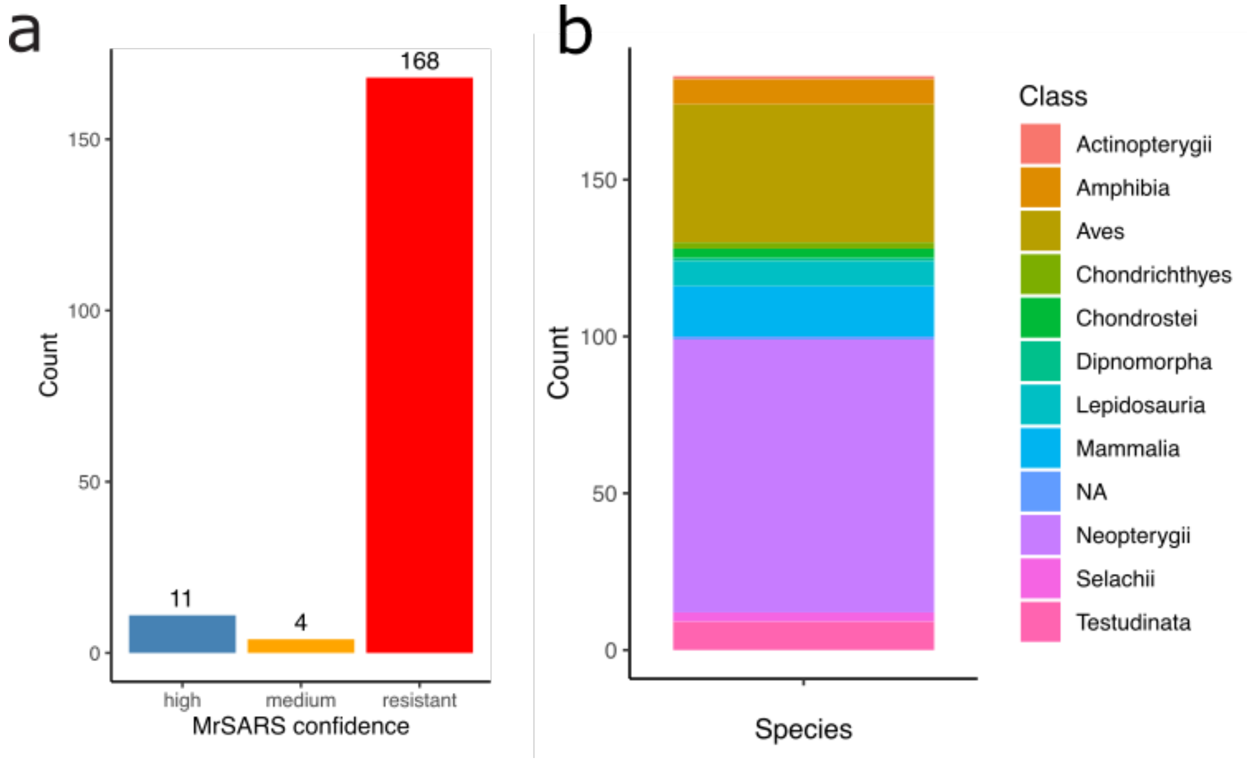

Figure S8: Susceptibility and phylogenetic summary of sequences uniquely analyzed by MrSARS. Counts of ACE2 sequences analyzed with MrSARS and not reported in the literature are presented according to their MrSARS confidence category **(a)** and phylogenetic group **(b)**.

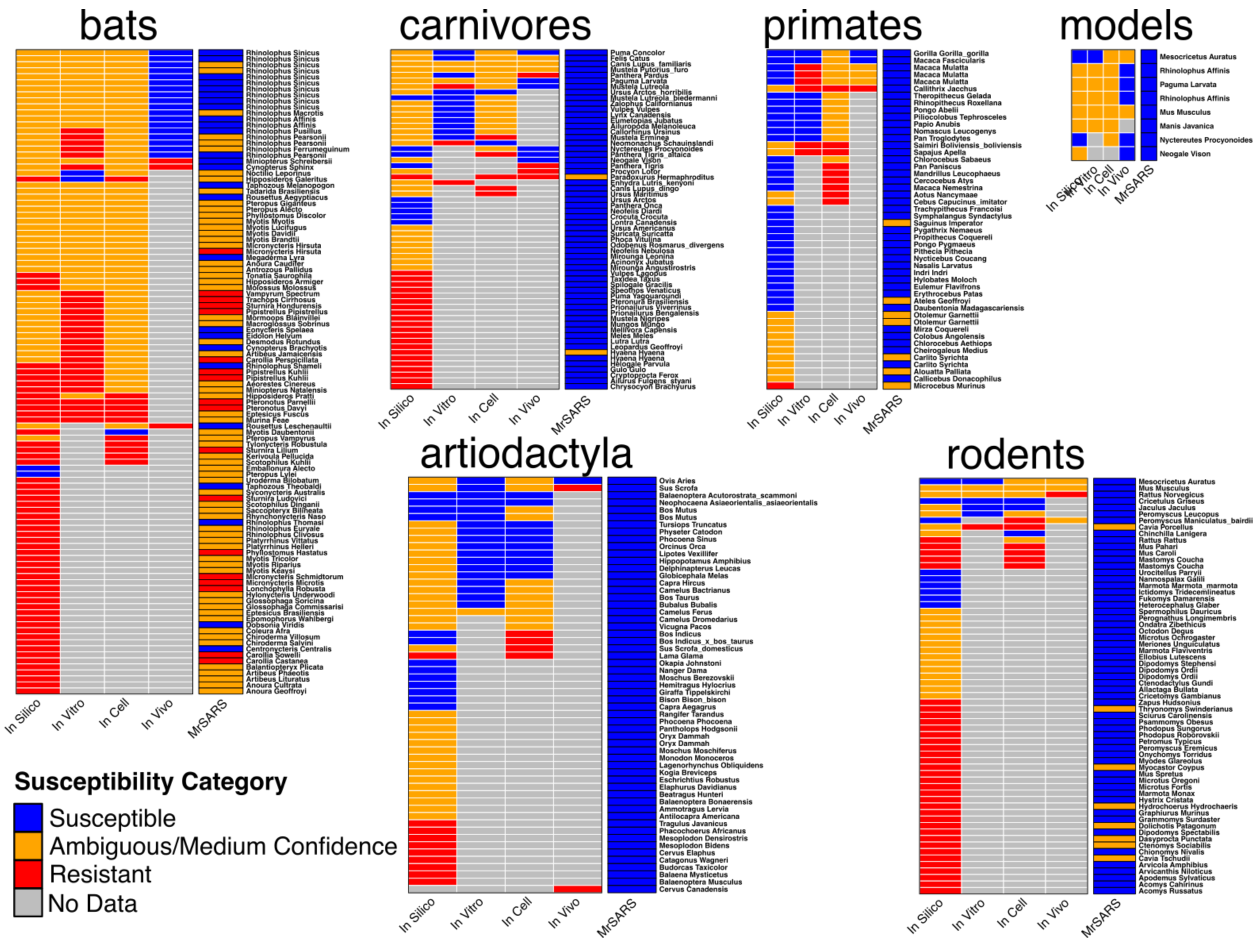

Figure S9: Lineage-specific MrSARS predictions compared to literature organized by study type Heatmaps summarize mammalian species susceptibility to SARS-CoV/SARS-CoV-2-related sarbecoviruses. Left four columns of heat maps depict literature support for susceptibility of indicated mammalian orders. Literature was categorized by in silico, in vitro, in cellus, and in vivo studies. Heatmap is colored according to literature and MrSARS susceptibility categories. Gray boxes represent the absence of studies affirming ACE2 usage in the given study type. Duplicate species represent distinct ACE2 allele susceptibility predictions made by MrSARS. See supplemental Table S1 for a complete summary of screened literature.

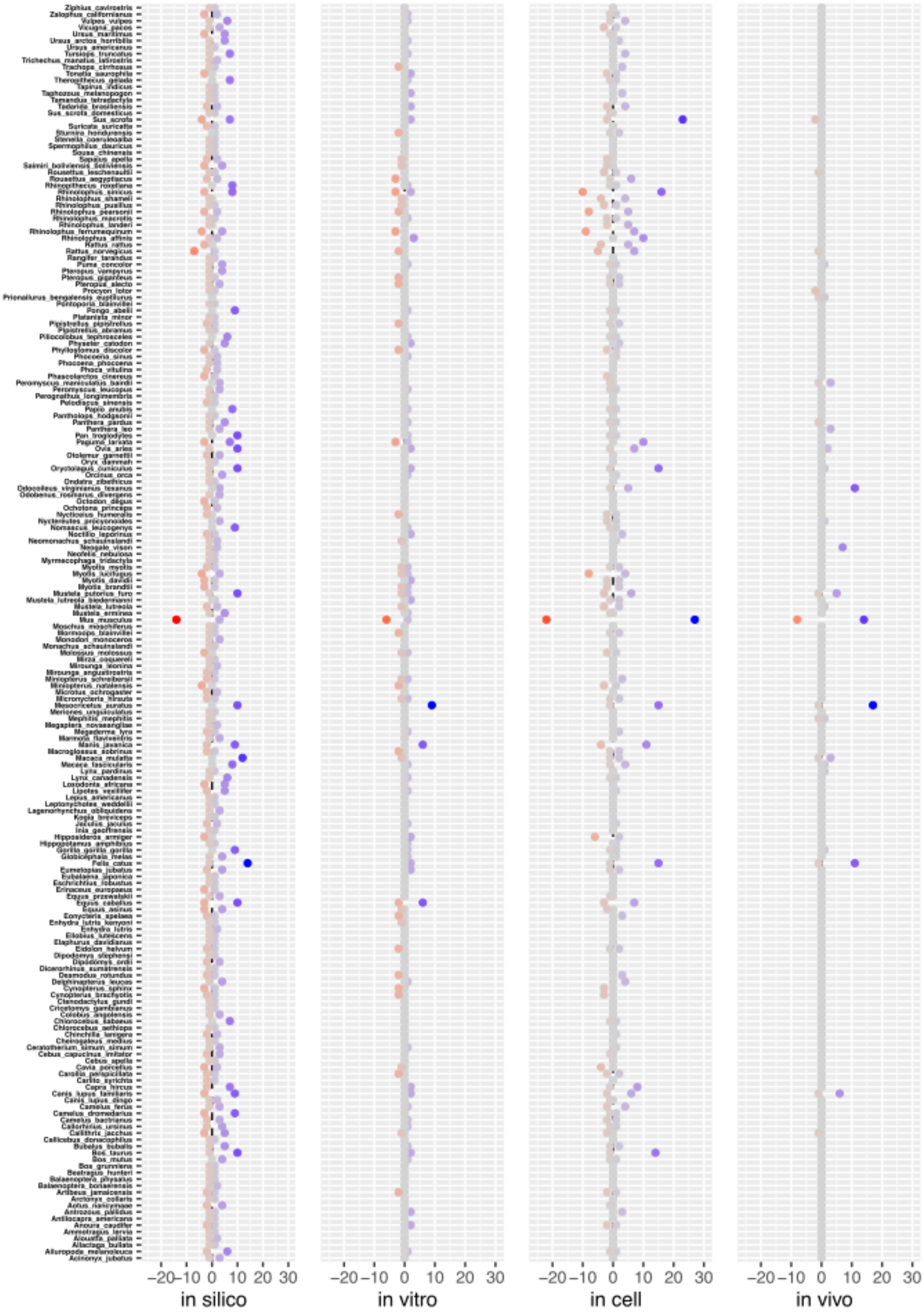

Figure S10: Literature conflict for animal susceptibility to SARS-CoV-2. Dot plots denote the number of studies that report species susceptibility (+ values, blue) or resistance (- values, red) to SARS-CoV-2. Dot color intensity correlates with the number of susceptibility and resistance reports. No reports for any given category are presented as gray dots with a value of 0.

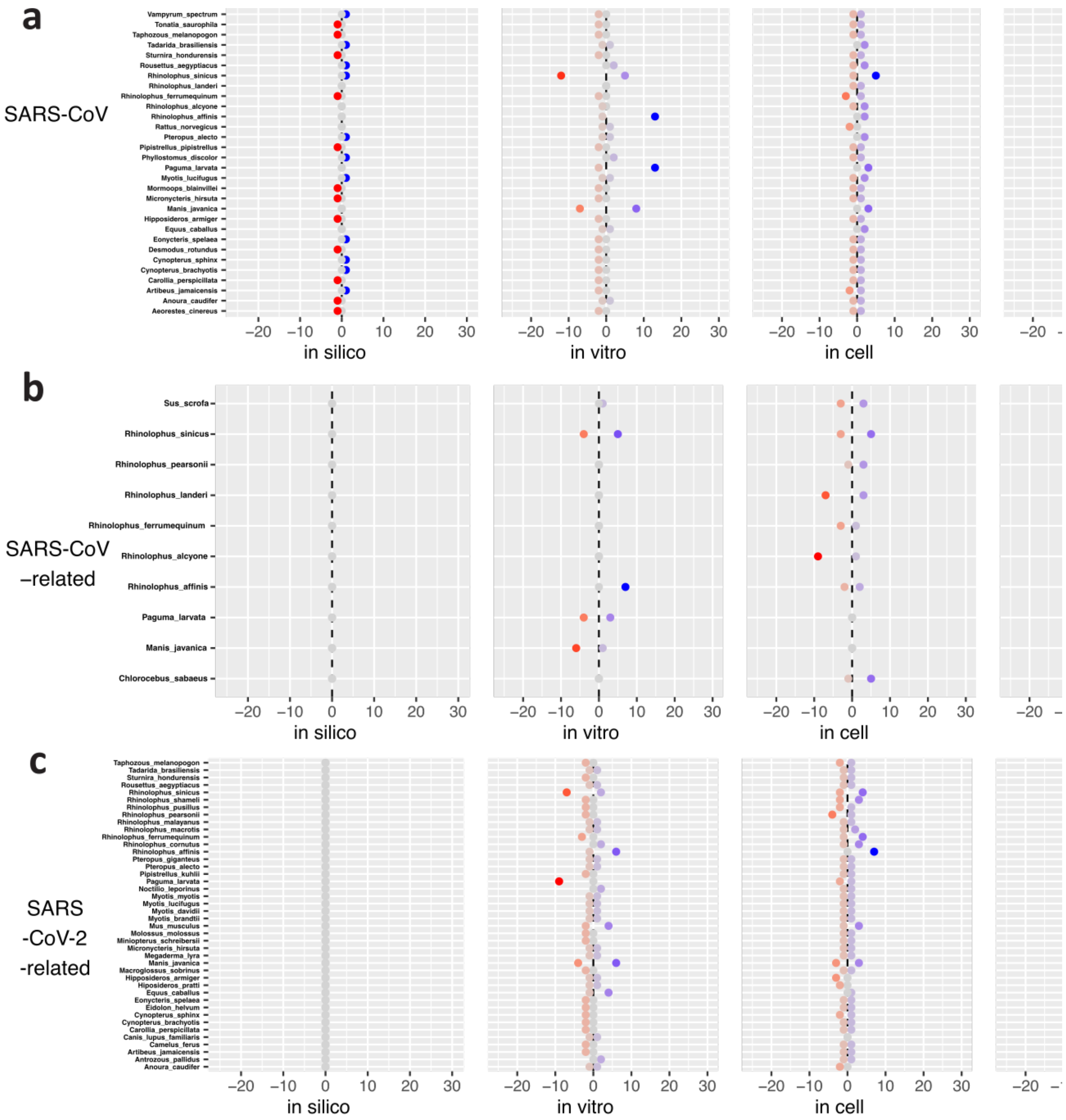

Figure S11: Literature conflict for animal susceptibility to SARS-CoV-related sarbecoviruses. a-c) Dot plots denote the number of studies that report species susceptibility (+ values, blue) or resistance (- values, red) to SARS-CoV (a), SARS-CoV-related (b), and SARS-CoV-2-related (c) sarbecoviruses. Dot color intensity correlates with the number of susceptibility and resistance reports. No reports for a given category are presented as gray dots with a value of 0.

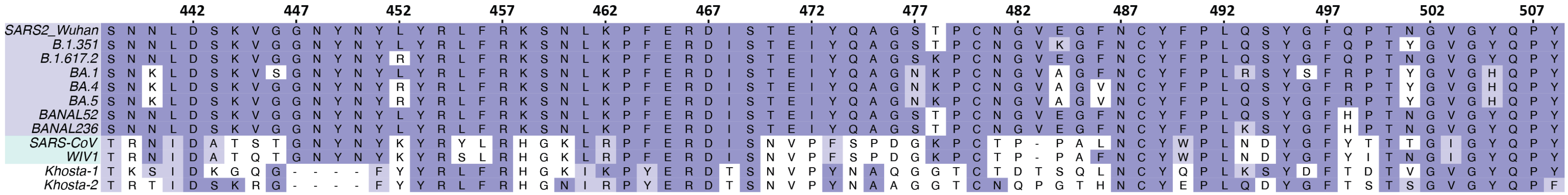
442
447
452
457
462
467
472
477
482
487
492
497
502
507
SARS2_Wuhan
B.1.351
B.1.617.2
BA.1
BA.4
BA.5
BANAL52
BANAL236
SARS-CoV
WIV1
Khosta-1
Khosta-2

Figure S12: Sequence alignment of ACE2-bound spike residues from indicated sarbecovirus strains. Residues are colored according to jalview-defined BLOSUM62 sequence similarity.
